# A Strong Converse Theorem for Hypothesis Testing Against Independence over a Two-Hop Network †

**DOI:** 10.3390/e21121171

**Published:** 2019-11-29

**Authors:** Daming Cao, Lin Zhou, Vincent Y. F. Tan

**Affiliations:** 1 School of Information Science and Engineering, Southeast University, Nanjing 210000, China; dmcao@seu.edu.cn; 2 Department of Electrical Engineering and Computer Science, University of Michigan, Ann Arbor, MI 48109, USA; 3 Department of Electrical and Computer Engineering and Department of Mathematics, National University of Singapore, Singapore 119077, Singapore; vtan@nus.edu.sg

**Keywords:** strong converse, hypothesis testing with communication constraints, testing against independence, two-hop network, relay

## Abstract

By proving a strong converse theorem, we strengthen the weak converse result by Salehkalaibar, Wigger and Wang (2017) concerning hypothesis testing against independence over a two-hop network with communication constraints. Our proof follows by combining two recently-proposed techniques for proving strong converse theorems, namely the strong converse technique via reverse hypercontractivity by Liu, van Handel, and Verdú (2017) and the strong converse technique by Tyagi and Watanabe (2018), in which the authors used a change-of-measure technique and replaced hard Markov constraints with soft information costs. The techniques used in our paper can also be applied to prove strong converse theorems for other multiterminal hypothesis testing against independence problems.

## 1. Introduction

Motivated by situations where the source sequence is *not* available directly and can only be obtained through limited communication with the data collector, Ahlswede and Csiszár [1] proposed the problem of hypothesis testing with a communication constraint. In the setting of [1], there is one encoder and one decoder. The encoder has access to one source sequence $X^{n}$ and transmits a compressed version of it to the decoder at a limited rate. Given the compressed version and the available source sequence $Y^{n}$ (side information), the decoder knows that the pair of sequences $\left(X^{n},Y^{n}\right)$ is generated i.i.d. from one of the two distributions and needs to determine which distribution the pair of sequences is generated from. The goal in this problem is to study the tradeoff between the compression rate and the exponent of the type-II error probability under the constraint that the type-I error probability is either vanishing or non-vanishing. For the special case of testing against independence, Ahlswede and Csiszár provided an exact characterization of the rate-exponent tradeoff. They also derived the so-called strong converse theorem for the problem. This states that the rate-exponent tradeoff cannot be improved even when one is allowed a non-vanishing type-I error probability. However, the characterization the rate-exponent tradeoff for the general case (even in the absence of a strong converse) remains open till date.

Subsequently, the work of Ahlswede and Csiszár was generalized to the distributed setting by Han in [2] who considered hypothesis testing over a Slepian-Wolf network. In this setting, there are two encoders, each of which observes one source sequence and transmits a compressed version of the source to the decoder. The decoder then performs a hypothesis test given these two compression indices. The goal in this problem is to study the tradeoff between the coding rates and the exponent of type-II error probability, under the constraint that the type-I error probability is either vanishing or non-vanishing. Han derived an inner bound to the rate-exponent region. For the special case of zero-rate communication, Shalaby and Papamarcou [3] applied the blowing-up lemma [4] judiciously to derive the exact rate-exponent region and a strong converse theorem. Further generalizations of the work of Ahlswede and Csiszár can be categorized into two classes: non-interactive models where encoders do not communicate with one another [5,6,7,8] and the interactive models where encoders do communicate [9,10].

**Figure 1:**
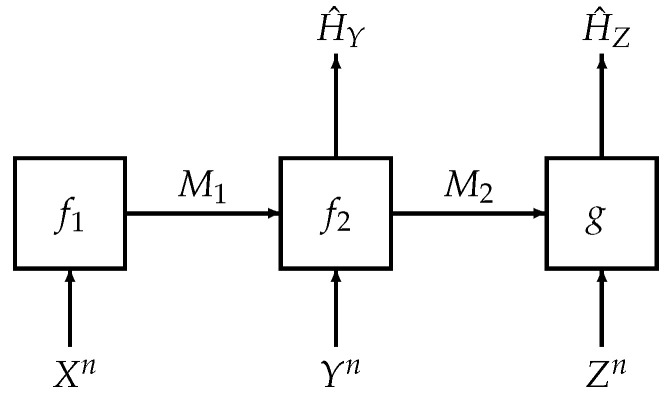
System model for hypothesis testing over a two-hop network

We revisit one such interactive model as shown in Figure 1. This problem was considered by Salehkalaibar, Wigger and Wang in [11] and we term the problem as *hypothesis testing over a two-hop network*. The two-hop model considered here has potential applications in the Internet of Things (IoT) and sensor networks. In these scenarios, direct communication from the transmitter to the receiver might *not* be possible due to power constraints that result from limited resources such as finite battery power. However, it is conceivable in such a scenario to assume that there are relays—in our setting, there is a single relay—that aid in the communication or other statistical inference tasks (such as hypothesis testing) between the transmitter and receiver.

The main task in this problem is to construct two hypothesis tests between two joint distributions $P_{XYZ}$ and $Q_{XYZ}$. One of these two distributions governs the law of $\left(X^{n},Y^{n},Z^{n}\right)$ where each copy $\left(X_{i},Y_{i},Z_{i}\right)$ is generated independently either from $P_{XYZ}$ and $Q_{XYZ}$. As shown in Figure 1, the first terminal has knowledge of a source sequence $X^{n}$ and sends an index $M_{1}$ to the second terminal, which we call the relay; the relay, given side information $Y^{n}$ and compressed index $M_{1}$, makes a guess of the hypothesis ${\hat{H}}_{Y}$ and sends another index $M_{2}$ to the third terminal; the third terminal makes another guess of the hypothesis ${\hat{H}}_{Z}$ based on $M_{2}$ and its own side information $Z^{n}$. The authors in [11] derived an inner bound for the rate-exponent region and showed that the bound is tight for several special cases, including the case of testing against independence in which $Q_{XYZ}=P_{X}P_{Y}P_{Z}$. However, even in this simpler case of testing against independence, which is our main concern in this paper, the authors in [11] *only* established a *weak* converse.

In this paper, we strengthen the result by Salehkalaibar, Wigger and Wang in [11] by deriving a *strong* converse for the case of testing against independence. Our proof follows by combining two recently proposed strong converse techniques by Liu et al. in [12] and by Tyagi and Watanabe in [13]. In [12], the authors proposed a framework to prove strong converse theorems based on functional inequalities and reverse hypercontractivity of Markov semigroups. In particular, they applied their framework to derive strong converse theorems for a collection of problems including the hypothesis testing with communication constraints problem in [1]. In [13], the authors proposed another framework for strong converse proofs, where they used a change-of-measure technique and replaced hard Markov constraints with soft information costs. They also leveraged variational formulas for various information-theoretic quantities; these formulas were introduced by Oohama in [14,15,16].

### Notation

Random variables and their realizations are in upper (e.g., *X*) and lower case (e.g., *x*) respectively. All sets are denoted in calligraphic font (e.g., X). We use $X^{c}$ to denote the complement of X. Let $X^{n}:=\left(X_{1},\ldots ,X_{n}\right)$ be a random vector of length *n* and $x^{n}$ its realization. Given any $x^{n}$, we use ${\hat{P}}_{x^{n}}$ to denote its type (empirical distribution). All logarithms are base *e*. We use $R_{+}$ and N to denote the set of non-negative real numbers and natural numbers respectively. Given any positive integer a∈N, we use [a] to denote {1,⋯,a}. We use 1{·} to denote the indicator function and use standard asymptotic notation such as O(·). The set of all probability distributions on a finite set X is denoted as P(X). Given any two random variables (X,Y) and any realization of *x*, we use $P_{Y|x}\left(\cdot \right)$ to denote the conditional distribution $P_{Y|X}\left(\cdot |x\right)$. Given a distribution P∈P(X) and a function f:X→R, we use P(f) to denote $E_{P}\left[f\left(X\right)\right]$. For information-theoretic quantities, we follow [17]. In particular, when the joint distribution of (X,Y) is $P_{XY}\in P\left(X\times Y\right)$, we use $I_{P_{XY}}\left(X;Y\right)$ and $I_{P}\left(X;Y\right)$ interchangeably. Throughout the paper, for ease of notation, we drop the subscript for distributions when there is no confusion. For example, when the joint distribution of (X,Y,Z) is $P_{XYZ}$, we use $I_{P}\left(X;Y|Z\right)$ and $I_{P_{XYZ}}\left(X;Y|Z\right)$ interchangeably. For any $\left(p,q\right)\in {\left[0,1\right]}^{2}$, let $D_{b}\left(p∥q\right)$ denote the binary divergence function, i.e., $D_{b}\left(p∥q\right)=p\log\left(p/q\right)+\left(1-p\right)\log\left(\left(1-p\right)/\left(1-q\right)\right)$.

## 2. Problem Formulation and Existing Results

### 2.1. Problem Formulation

Fix a joint distribution $P_{XYZ}\in P\left(X\times Y\times Z\right)$ satisfying the Markov chain X−Y−Z, i.e.,

(1) $$\begin{array}{c} P_{XYZ}\left(x,y,z\right)=P_{X}\left(x\right)P_{Y|X}\left(y|x\right)P_{Z|Y}\left(z|y\right). \end{array}$$

Let $P_{X}$, $P_{Y}$ and $P_{Z}$ be induced marginal distributions of $P_{XYZ}$. As shown in Figure 1, we consider a two-hop hypothesis testing problem with three terminals. The first terminal, which we term the transmitter, observes a source sequence $X^{n}$ and sends a compression index $M_{1}$ to the second terminal, which we term the relay. Given $M_{1}$ and side information $Y^{n}$, the relay sends another compression index $M_{2}$ to the third terminal, which we term the receiver. The main task in this problem is to construct hypothesis tests at both the relay and the receiver to distinguish between

(2) $$\begin{array}{c} H_{0}:\left(X^{n},Y^{n},Z^{n}\right)∼P_{XYZ}^{n}=P_{X}P_{Y|X}^{n}P_{Z|Y}^{n}, \end{array}$$

(3) $$\begin{array}{c} H_{1}:\left(X^{n},Y^{n},Z^{n}\right)∼P_{X}^{n}P_{Y}^{n}P_{Z}^{n}. \end{array}$$

For subsequent analyses, we formally define a code for hypothesis testing over a two-hop network as follows.

**Definition** **1.** *An $\left(n,N_{1},N_{2}\right)$-code for hypothesis testing over a two-hop network consists of*

- *Two encoders:*
  (4) $$\begin{array}{cc} f_{1} & :X^{n}\to M_{1}:=\left\{1,\ldots ,N_{1}\right\}, \end{array}$$
  (5) $$\begin{array}{cc} f_{2} & :M_{1}\times Y^{n}\to M_{2}:=\left\{1,\ldots ,N_{2}\right\},\;\mathrm{and} \end{array}$$
- *Two decoders*
  (6) $$\begin{array}{cc} g_{1} & :M_{1}\times Y^{n}\to \left\{H_{0},H_{1}\right\}, \end{array}$$
  (7) $$\begin{array}{cc} g_{2} & :M_{2}\times Z^{n}\to \left\{H_{0},H_{1}\right\}. \end{array}$$

Given an $\left(n,N_{1},N_{2}\right)$-code with encoding and decoding functions $\left(f_{1},f_{2},g_{1},g_{2}\right)$, we define acceptance regions for the null hypothesis $H_{0}$ at the relay and the receiver as

(8) $$\begin{array}{cc} A_{Y,n} & :=\{\left(m_{1},y^{n}\right):g_{1}\left(m_{1},y^{n}\right)=H_{0}\}, \end{array}$$

(9) $$\begin{array}{cc} A_{Z,n} & :=\{\left(m_{2},z^{n}\right):g_{2}\left(m_{2},z^{n}\right)=H_{0}\} \end{array}$$

respectively. We also define conditional distributions

(10) $$\begin{array}{cc} P_{M_{1}|X^{n}}\left(m_{1}|x^{n}\right) & :=1\{f_{1}\left(x_{1}^{n}\right)=m_{1}\}, \end{array}$$

(11) $$\begin{array}{cc} P_{M_{2}|Y^{n}M_{1}}\left(m_{2}|y^{n},m_{1}\right) & :=1\{f_{2}\left(m_{1},y^{n}\right)=m_{2}\}. \end{array}$$

Thus, for a $\left(n,N_{1},N_{2}\right)$-code characterized by $\left(f_{1},f_{2},g_{1},g_{2}\right)$, the joint distribution of random variables $\left(X^{n},Y^{n},Z^{n},M_{1},M_{2}\right)$ under the null hypothesis $H_{0}$ is given by

(12) $$\begin{array}{cc} P_{X^{n}Y^{n}Z^{n}M_{1}M_{2}}\left(x^{n},y^{n},z^{n},m_{1},m_{2}\right) & =P_{XYZ}^{n}\left(x^{n},y^{n},z^{n}\right)P_{M_{1}|X^{n}}\left(m_{1}|x^{n}\right)P_{M_{2}|Y^{n}M_{1}}\left(m_{2}|y^{n},m_{1}\right), \end{array}$$

and under the alternative hypothesis $H_{1}$ is given by

(13) $$\begin{array}{c} {\bar{P}}_{X^{n}Y^{n}Z^{n}M_{1}M_{2}}\left(x^{n},y^{n},z^{n},m_{1},m_{2}\right)=P_{X}^{n}\left(x^{n}\right)P_{Y}^{n}\left(y^{n}\right)P_{Z}^{n}\left(z^{n}\right)P_{M_{1}|X^{n}}\left(m_{1}|x^{n}\right)P_{M_{2}|Y^{n}M_{1}}\left(m_{2}|y^{n},m_{1}\right). \end{array}$$

Now, let $P_{Y^{n}M_{1}}$ and $P_{Z^{n}M_{2}}$ be marginal distributions induced by $P_{X^{n}Y^{n}Z^{n}M_{1}M_{2}}$ and let ${\bar{P}}_{Y^{n}M_{1}}$ and ${\bar{P}}_{Z^{n}M_{2}}$ be marginal distributions induced by ${\bar{P}}_{X^{n}Y^{n}Z^{n}M_{1}M_{2}}$. Then, we can define the type-I and type-II error probabilities at the relay as

(14) $$\begin{array}{cc} \beta_{1} & :=P_{M_{1}Y^{n}}\left(A_{Y,n}^{c}\right), \end{array}$$

(15) $$\begin{array}{cc} \beta_{2} & :={\bar{P}}_{M_{1}Y^{n}}\left(A_{Y,n}\right) \end{array}$$

respectively and at the receiver as

(16) $$\begin{array}{cc} \eta_{1} & :=P_{M_{2}Z^{n}}\left(A_{Z,n}^{c}\right), \end{array}$$

(17) $$\begin{array}{cc} \eta_{2} & :={\bar{P}}_{M_{2}Z^{n}}\left(A_{Z,n}\right) \end{array}$$

respectively. Clearly, $\beta_{1},\beta_{2},\eta_{1}$, and $\eta_{2}$ are functions of *n* but we suppress these dependencies for brevity.

Given above definitions, the achievable rate-exponent region for the hypothesis testing problem in a two-hop network is defined as follows.

**Definition** **2.** *Given any $\left(\varepsilon_{1},\varepsilon_{2}\right)\in {\left(0,1\right)}^{2}$, a tuple $\left(R_{1},R_{2},E_{1},E_{2}\right)$ is said to be* $\left(\varepsilon_{1},\varepsilon_{2}\right)$ *-achievable if there exists a sequence of $\left(n,N_{1},N_{2}\right)$-codes such that*

(18) $$\begin{array}{cc} \underset{n\to \infty }{lim sup}\frac{1}{n}\log N_{i} & \leq R_{i},\;\forall i\in \left\{1,2\right\}, \end{array}$$

(19) $$\begin{array}{cc} \underset{n\to \infty }{lim sup}\beta_{1} & \leq \varepsilon_{1}, \end{array}$$

(20) $$\begin{array}{cc} \underset{n\to \infty }{lim sup}\eta_{1} & \leq \varepsilon_{2}, \end{array}$$

(21) $$\begin{array}{cc} \underset{n\to \infty }{lim inf}-\frac{1}{n}\log\beta_{2} & \geq E_{1}, \end{array}$$

(22) $$\begin{array}{cc} \underset{n\to \infty }{lim inf}-\frac{1}{n}\log\eta_{2} & \geq E_{2}. \end{array}$$

*The closure of the set of all $\left(\varepsilon_{1},\varepsilon_{2}\right)$-achievable rate-exponent tuples is called the* $\left(\varepsilon_{1},\varepsilon_{2}\right)$ *-rate-exponent region and is denoted as $R(\varepsilon_{1},\varepsilon_{2})$. Furthermore, define the rate-exponent region as*

(23) $$\begin{array}{cc} R & :=R(0,0). \end{array}$$

### 2.2. Existing Results

In the following, we recall the exact characterization of R given by Salehkalaibar, Wigger and Wang ([11] (Corollary 1)). For this purpose, define the following set of joint distributions

(24) $$\begin{array}{c} Q:=\{Q_{XYZUV}\in P\left(X\times Y\times Z\times U\times V\right):Q_{XYZ}=P_{XYZ},\;U-X-Y,\;V-Y-Z\}. \end{array}$$

Given $Q_{XYZUV}\in Q$, define the following set

(25) $$\begin{array}{cc} R\left(Q_{XYZUV}\right):=\{\left(R_{1},R_{2},E_{1},E_{2}\right):R_{1} & \geq I_{Q}\left(U;X\right),R_{2}\geq I_{Q}\left(V;Y\right),\\ E_{1} & \leq I_{Q}\left(U;Y\right),E_{2}\leq I_{Q}\left(U;Y\right)+I_{Q}\left(V;Z\right)\} \end{array}$$

Finally, let

(26) $$\begin{array}{cc} R^{*} & :=\underset{Q_{XYZUV}\in Q}{⋃}R\left(Q_{XYZUV}\right). \end{array}$$

**Theorem** **1.** *The rate-exponent region R for the hypothesis testing over a two-hop network problem satisfies*

(27) $$\begin{array}{c} R=R^{*}. \end{array}$$

In the following, inspired by Oohama’s variational characterization of rate regions for multiuser information theory [14,15,16], we provide an alternative characterization of $R^{*}$. For this purpose, given any $\left(b,c,d\right)\in R_{+}^{3}$ and any $Q_{XYZUV}\in Q$, let

(28) $$\begin{array}{cc} R_{b,c,d}\left(Q_{XYZUV}\right) & :=-I_{Q}\left(U;Y\right)+bI_{Q}\left(U;X\right)-c\left(I_{Q}\left(U;Y\right)+I_{Q}\left(V;Z\right)\right)+dI_{Q}\left(V;Y\right). \end{array}$$

be a linear combination of the mutual information terms in (25). Furthermore, define

(29) $$\begin{array}{cc} R_{b,c,d} & :=\min_{Q_{XYZUV}\in Q}R_{b,c,d}\left(Q_{XYZUV}\right). \end{array}$$

An alternative characterization of $R^{*}$ is given by

(30) $$\begin{array}{c} R^{*}=\underset{\left(b,c,d\right)\in R_{+}^{3}}{⋂}\left\{\left(R_{1},R_{2},E_{1},E_{2}\right):-E_{1}+bR_{1}-cE_{2}+dR_{2}\geq R_{b,c,d}\right\}. \end{array}$$

## 3. Strong Converse Theorem

### 3.1. The Case $\varepsilon_{1}+\varepsilon_{2}<1$

**Theorem** **2.** *Given any $\left(\varepsilon_{1},\varepsilon_{2}\right)\in {\left(0,1\right)}^{2}$ such that $\varepsilon_{1}+\varepsilon_{2}<1$ and any $\left(b,c,d\right)\in R_{+}^{3}$, for any $\left(n,N_{1},N_{2}\right)$-code such that $\beta_{1}\leq \varepsilon_{1}$, $\eta_{1}\leq \varepsilon_{2}$, we have*

(31) $$\begin{array}{c} \log\beta_{2}+b\log N_{1}+c\log\eta_{2}+d\log N_{2}\geq nR_{b,c,d}+\Theta \left(n^{3/4}\log n\right). \end{array}$$

The proof of Theorem 2 is given in Section 4. Several remarks are in order.

First, using the alternative expression of the rate-exponent region in (30), we conclude that for any $\left(\varepsilon_{1},\varepsilon_{2}\right)\in {\left(0,1\right)}^{2}$ such that $\varepsilon_{1}+\varepsilon_{2}<1$, we have $R\left(\varepsilon_{1},\varepsilon_{2}\right)=R^{*}$. This result significantly strengthens the weak converse result in ([11] (Corollary 1)) in which it was shown that $R\left(0,0\right)=R^{*}$.

Second, it appears difficult to establish the strong converse result in Theorem 2 using existing classical techniques including image-size characterizations (a consequence of the blowing-up lemma) [4,6] and the perturbation approach [18]. In Section 4, we combine two recently proposed strong converse techniques by Liu, van Handel, and Verdú [12] and by Tyagi and Watanabe [13]. In particular, we use the strong converse technique based on reverse hypercontractivity in [12] to bound the exponent of the type-II error probability at the receiver and the strong converse technique in [13], which leverages an appropriate change-of-measure technique and replaces hard Markov constraints with soft information costs, to analyze the exponent of type-II error probability at the relay. Finally, inspired by the single-letterization steps in ([19] (Lemma C.2)) and [13], we single-letterize the derived multi-letter bounds from the previous steps to obtain the desired result in Theorem 2.

Third, we briefly comment on the apparent necessity of combining the two techniques in [12,13] instead of applying just one of them to obtain Theorem 2. The first step to apply the technique in [13] is to construct a “truncated source distribution” which is supported on a smaller set (often defined in terms of the decoding region) and is not too far away from the true source distribution in terms of the relative entropy. For our problem, the source satisfies the Markov chain $X^{n}-Y^{n}-Z^{n}$. If we naïvely apply the techniques in [13], the Markovian property would not hold for the truncated source $\left({\tilde{X}}^{n},{\tilde{Y}}^{n},{\tilde{Z}}^{n}\right)$. On the other hand, it appears rather challenging to extend the techniques in [12] to the hypothesis testing over a multi-hop network problem since the techniques therein rely heavily on constructing semi-groups and it is difficult to devise appropriate forms of such semi-groups to be used and analyzed in this multi-hop setting. Therefore, we carefully combine the two techniques in [12,13] to ameliorate the aforementioned problems. In particular, we first use the technique in [13] to construct a truncated source $\left({\tilde{X}}^{n},{\tilde{Y}}^{n}\right)$ and then let the conditional distribution of ${\tilde{Z}}^{n}$ given $\left({\tilde{X}}^{n},{\tilde{Y}}^{n}\right)$ be given by the *true* conditional source distribution $P_{Z|Y}^{n}$ to maintain the Markovian property of the source (see (56)). Subsequently, in the analysis of error exponents, we use the technique in [12] to analyze the exponent of type-II error probability at the receiver to circumvent the need to construct new semi-groups.

Finally, we remark that the techniques (or a subset of the techniques) used to prove Theorem 2 can also be used to establish a strong converse result for other multiterminal hypothesis testing against independence problems, e.g., hypothesis testing over the Gray-Wyner network [7], the interactive hypothesis testing problem [9] and the cascaded hypothesis testing problem [10].

### 3.2. The Case $\varepsilon_{1}+\varepsilon_{2}>1$

In this subsection, we consider the case where the sum of type-I error probabilities at the relay and the receiver is upper bounded by a quantity strictly greater than one. For ease of presentation of our results, let

(32) $$\begin{array}{cc} Q_{2} & :=\{Q_{XYZU_{1}U_{2}V}\in Q\left(X\times Y\times Z\times U_{1}\times U_{2}\times V\right):\\ & Q_{XYZ}=P_{XYZ},U_{1}-X-Y,\;U_{2}-X-Y,\;V-Y-Z\}. \end{array}$$

Given any $Q_{XYZU_{1}U_{2}V}\in Q_{2}$, define the following set of rate-exponent tuples

(33) $$\begin{array}{cc} \tilde{R}\left(Q_{XYZU_{1}U_{2}V}\right):=\{\left(R_{1},R_{2},E_{1},E_{2}\right): & R_{1}\geq \max\left\{I_{Q}\left(U_{1};X\right),I_{Q}\left(U_{2};X\right)\right\},\;R_{2}\geq I_{Q}\left(V;X\right),\\ & E_{1}\leq I_{Q}\left(U_{1};Y\right),\;E_{2}\leq I_{Q}\left(U_{2};Y\right)+I_{Q}\left(V;Z\right)\}. \end{array}$$

Furthermore, define

(34) $$\begin{array}{cc} \tilde{R} & :=\underset{Q_{XYZU_{1}U_{2}V}}{⋃}\tilde{R}\left(Q_{XYZU_{1}U_{2}V}\right). \end{array}$$

Given any $Q_{XYZU_{1}U_{2}V}\in Q_{2}$ and $\left(b_{1},b_{2},c,d\right)\in R_{+}^{4}$, define the following linear combination of the mutual information terms

(35) $$\begin{array}{c} {\tilde{R}}_{b_{1},b_{2},c,d}\left(Q_{XYZU_{1}U_{2}V}\right)\\ :=-I_{Q}\left(U_{1};Y\right)+b_{1}I_{Q}\left(U_{1};X\right)+b_{2}I_{Q}\left(U_{2};X\right)-c\left(I_{Q}\left(U_{2};Y\right)+I_{Q}\left(V;Z\right)\right)+dI_{Q}\left(V;Y\right), \end{array}$$

and let

(36) $$\begin{array}{cc} {\tilde{R}}_{b_{1},b_{2},c,d} & :=\min_{Q_{XYZU_{1}U_{2}V}}{\tilde{R}}_{b_{1},b_{2},c,d}\left(Q_{XYZU_{1}U_{2}V}\right). \end{array}$$

Then, based on [14,15,16], an alternative characterization of $\tilde{R}$ is given by

(37) $$\begin{array}{cc} \tilde{R} & =\underset{\left(b_{1},b_{2},c,d\right)\in R_{+}^{4}}{⋃}\left\{\left(R_{1},R_{2},E_{1},E_{2}\right):-E_{1}+b_{1}R_{1}+b_{2}R_{1}-cE_{2}+dR_{2}\geq {\tilde{R}}_{b_{1},b_{2},c,d}\right\}. \end{array}$$

Analogously to Theorem 2, we obtain the following result.

**Theorem** **3.** *Given any $\left(\varepsilon_{1},\varepsilon_{2}\right)\in {\left(0,1\right)}^{2}$ and any $\left(b_{1},b_{2},c,d\right)\in R_{+}^{4}$, for any $\left(n,N_{1},N_{2}\right)$-code such that $\beta_{1}\leq \varepsilon_{1}$, $\eta_{1}\leq \varepsilon_{2}$, we have*

(38) $$\begin{array}{c} \log\beta_{2}+b_{1}\log N_{1}+b_{2}\log N_{1}+c\log\eta_{2}+d\log N_{2}\geq n{\tilde{R}}_{b_{1},b_{2},c,d}+\Theta \left(n^{3/4}\log n\right). \end{array}$$

The proof of Theorem 3 is similar to that of Theorem 2 and thus omitted for simplicity.

**Figure 2:**
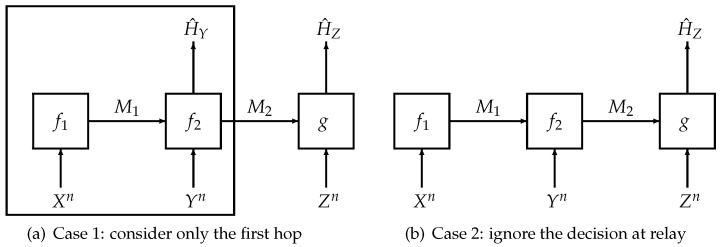
Illustration of the proof sketch of Theorem 3.

To prove Theorem 3, we need to analyze two special cases (cf. Figure 2) of our system model separately:

(i) Firstly, we consider the first hop, which involves the transmitter and the relay only. The first hop itself is a hypothesis testing problem with a communication constraint [1]. Using the techniques either in [13] or [12], we can obtain bounds on a linear combination of the rate of the first encoder and the type-II error exponent of the relay, (i.e., $\log\beta_{2}+b_{1}\log N_{1}$ for any $b_{1}\in R_{+}$) for any type-I error probability $\beta_{1}\in \left(0,1\right)$ at the relay.
(ii) Secondly, we study the second special case in which the relay does not make a decision. Using similar steps to the proof of Theorem 2, we can obtain a lower bound on a linear combination of the rate at the transmitter, the rate at the relay and the type-II exponent at the receiver (i.e., $b_{2}\log N_{1}+c\log\eta_{2}+d\log N_{2}$ for any $\left(b_{2},c,d\right)\in R_{+}^{3}$) for any type-I error probability $\eta_{1}\in \left(0,1\right)$ at the receiver.
(iii) Finally, combining the results obtained in the first two steps, we obtain a lower bound on the linear combination of rates and type-II exponents (as shown in Theorem 3). The proof is completed by using standard single-letterization steps and the variational formula in Equation (37).

Using Theorem 3, we obtain the following proposition.

**Proposition** **1.** *For any $\left(\varepsilon_{1},\varepsilon_{2}\right)\in {\left(0,1\right)}^{2}$ such that $\varepsilon_{1}+\varepsilon_{2}>1$, we have*

(39) $$\begin{array}{c} R\left(\varepsilon_{1},\varepsilon_{2}\right)=\tilde{R}. \end{array}$$

The converse proof of Proposition 1 follows from Theorem 3 and the alternative characterization of $\tilde{R}$ in (37). The achievability proof is inspired by ([6] (Theorem 5)) and is provided in Appendix A. The main idea is that we can time-share between two close-to optimal coding schemes, each of which corresponds to one special case of the current problem as mentioned after Theorem 3.

Recall that in the first remark of Theorem 2, we provide an exact characterization of the rate-exponent region for any $\left(\varepsilon_{1},\varepsilon_{2}\right)\in {\left(0,1\right)}^{2}$ such that $\varepsilon_{1}+\varepsilon_{2}<1$. The converse proof follows from Theorem 2 and the achievability part was given in ([20] (Corollary 1)). Combining the first remark of Theorem 2 and Proposition 1, we provide an exact characterization of $R(\varepsilon_{1},\varepsilon_{2})$ for any $\left(\varepsilon_{1},\varepsilon_{2}\right)\in {\left(0,1\right)}^{2}$ such that $\varepsilon_{1}+\varepsilon_{2}\neq 1$. We remark the case in which $\varepsilon_{1}+\varepsilon_{2}=1$ was also excluded in the analysis of the successive refinement of hypothesis testing with communication constraints problem studied by Tian and Chen [6]. In fact, our converse result in Theorem 3 holds for any $\left(\varepsilon_{1},\varepsilon_{2}\right)\in {\left(0,1\right)}^{2}$ including the case $\varepsilon_{1}+\varepsilon_{2}=1$. However, the achievability result presented in Appendix A holds only when $\varepsilon_{1}+\varepsilon_{2}>1$ and thus we are unable to characterize $R(\varepsilon_{1},\varepsilon_{2})$ when $\varepsilon_{1}+\varepsilon_{2}=1$. Because of the need to propose an achievability scheme which uses completely different techniques to handle the case in which $\varepsilon_{1}+\varepsilon_{2}=1$, which does not dovetail with the main message and contribution of this paper, we omit this case in this paper.

## 4. Proof of Theorem 2

### 4.1. Preliminaries

Before presenting the proof of Theorem 2, in this subsection, we briefly review the two strong converse techniques that we judiciously combine in this work, namely the change-of-measure technique by Tyagi and Watanabe [13] and the hypercontractivity technique by Liu et al. [12].

The critical step in the strong converse technique by Tyagi and Watanabe [13] is to construct a truncated source distribution, which is supported over a small set related to the decoding regions. Furthermore, the constructed truncated distribution should satisfy the following conditions:

(i) The truncated distribution is *close* to the original source distribution in terms of the KL divergence;
(ii) Under the truncated distribution, the (type-I) error probability is *small*.

Subsequent steps proceed similarly as the weak converse analysis of the problem and lead to bounds on the rates and (type-II) exponents. We then single-letterize the obtained bounds (using classical techniques in information theory without the memoryless property, e.g., [21]). Finally, we relate the single-letterized results to the the variational characterization [14,16] of the fundamental limit of the problem, which uses the idea of replacing hard Markov constraints with soft information costs.

The advantage of the Tyagi-Watanabe technique lies in its simplicity and similarity to weak converse analyses. In contrast, the disadvantage of the technique is that the structure of the source distribution (e.g., Markovian) is potentially *lost* in the constructed truncated distribution. As we have illustrated briefly after Theorem 2, this disadvantage prevents us from solely using the Tyagi-Watanabe technique to prove the strong converse theorem for our setting.

On the other hand, the key technique in the strong converse technique by Liu et al. [12] is the use of ideas from reverse hypercontractivity. In particular, one needs to use the variational formula of the KL divergence ([22] (Chapter 12)) and carefully construct Markov semigroups. The operation of applying a Markov semigroup is similar to a soft version of blowing up of decoding sets [4] for the discrete memoryless case. The advantage of the strong converse technique by Liu et al. lies in its wide applicability (beyond discrete settings) and its versatile performance (beyond showing strong converses it can be used to show that the second order terms scale as $O(\sqrt{n})$). However, the construction of appropriate Markov semigroups is problem-specific, which limits its applicability to other information-theoretic problems in the sense that one has to construct specific semigroups for each problem. Fortunately, in our setting this construction and combination with Tyagi-Watanabe’s technique, is feasible.

### 4.2. Summary of Proof Steps

In the rest of this section, we present the proof of strong converse theorem for the hypothesis testing over the two-hop network. The proof follows by combining the techniques in [12,13] and is separated into three main steps. First, we construct a truncated source distribution $P_{{\tilde{X}}^{n}{\tilde{Y}}^{n}{\tilde{Z}}^{n}}$ and show that this truncated distribution is not too different from $P_{XYZ}^{n}$ in terms of the relative entropy. Subsequently, we analyze the exponents of type-II error probabilities at the relay and the receiver under the constraint that their type-I error probabilities are non-vanishing. Finally, we single-letterize the constraints on rate and error exponents to obtain desired result in Theorem 2.

To begin with, let us fix an $\left(n,N_{1},N_{2}\right)$-code with functions $\left(f_{1},f_{2},g_{1},g_{2}\right)$ such that the type-I error probabilities are bounded above by $\varepsilon_{1}\in \left(0,1\right)$ and $\varepsilon_{2}\in \left(0,1\right)$ respectively, i.e., $\beta_{1}\leq \varepsilon_{1}$ and $\eta_{1}\leq \varepsilon_{2}$. We note from (19) and (20) that $\beta_{1}\leq \varepsilon_{1}+o\left(1\right)$ and $\beta_{2}\leq \varepsilon_{2}+o\left(1\right)$. Since the o(1) terms are immaterial in the subsequent analyses, they are omitted for brevity.

### 4.3. Step 1: Construction of a Truncated Distribution

Paralleling the definitions of acceptance regions in (8) and (9), we define the following acceptance regions at the relay and the receiver as

(40) $$\begin{array}{c} D_{Y,n}=\left\{\left(x^{n},y^{n}\right):g_{1}\left(f_{1}\left(x^{n}\right),y^{n}\right)=H_{0}\right\}, \end{array}$$

(41) $$\begin{array}{c} D_{Z,n}=\left\{\left(x^{n},y^{n},z^{n}\right):g_{2}\left(f_{2}\left(f_{1}\left(x^{n}\right),y^{n}\right),z^{n}\right)=H_{0}\right\}, \end{array}$$

respectively. Note that the only difference between $A_{Y,n}$ and $D_{Y,n}$ lies in whether we consider the compression index $m_{1}$ or the original source sequence $x^{n}$. Recalling the definitions of the type-I error probabilities for the relay denoted by $\beta_{1}$ in (14) and for the receiver denoted by $\eta_{1}$ in (16), and using (40) and (41), we conclude that

(42) $$\begin{array}{cc} P_{XY}^{n}\left(D_{Y,n}\right) & =1-\beta_{1}, \end{array}$$

(43) $$\begin{array}{cc} P_{XYZ}^{n}\left(D_{Z,n}\right) & =1-\eta_{1}. \end{array}$$

For further analysis, given any $m_{2}\in M_{2}$, define a conditional acceptance region at the receiver (conditioned on $m_{2}$) as

(44) $$\begin{array}{cc} G(m_{2}) & :=\{z^{n}:g_{2}\left(m_{2},z^{n}\right)=H_{0}\}. \end{array}$$

For ease of notation, given any $\left(x^{n},y^{n}\right)\in X^{n}\times Y^{n}$, we use $G(x^{n},y^{n})$ and $G(f_{2}\left(f_{1}\left(x^{n}\right),y^{n}\right))$ (here $f_{2}\left(f_{1}\left(x^{n}\right),y^{n}\right)$ plays the role of $m_{2}$ in (44)) interchangeably and define the following set

(45) $$\begin{array}{c} B_{n}:=\left\{\left(x^{n},y^{n}\right):P_{Z|Y}^{n}\left(G\left(x^{n},y^{n}\right)|y^{n}\right)\geq \frac{1-\varepsilon_{1}-\varepsilon_{2}}{1+3\varepsilon_{2}-\varepsilon_{1}}\right\}. \end{array}$$

Combining (41), (43) and (44), we obtain

(46) $$\begin{array}{cc} 1-\varepsilon_{2} & \leq P_{XYZ}^{n}\left(D_{Z,n}\right) \end{array}$$

(47) $$\begin{array}{cc} & =\sum_{\left(x^{n},y^{n}\right)\in B_{n}}P_{XY}^{n}\left(x^{n},y^{n}\right)P_{Z|Y}^{n}\left(G\left(x^{n},y^{n}\right)|y^{n}\right)+\sum_{\left(x^{n},y^{n}\right)\notin B_{n}}P_{XY}^{n}\left(x^{n},y^{n}\right)P_{Z|Y}^{n}\left(G\left(x^{n},y^{n}\right)|y^{n}\right) \end{array}$$

(48) $$\begin{array}{cc} & \leq P_{XY}^{n}\left(B_{n}\right)+\left(1-P_{XY}^{n}\left(B_{n}\right)\right)\frac{1-\varepsilon_{1}-\varepsilon_{2}}{1+3\varepsilon_{2}-\varepsilon_{1}}. \end{array}$$

Thus, we have

(49) $$\begin{array}{c} P_{XY}^{n}\left(B_{n}\right)\geq \frac{3-3\varepsilon_{2}+\varepsilon_{1}}{4}. \end{array}$$

For subsequent analyses, let

(50) $$\begin{array}{cc} \mu & :={\left(\min_{y:P_{Y}\left(y\right)>0}P_{Y}\left(y\right)\right)}^{-1}, \end{array}$$

(51) $$\begin{array}{cc} \theta_{n} & :=\sqrt{\frac{3\mu }{n}\log\frac{8|Y|}{1-\varepsilon_{1}-\varepsilon_{2}}}, \end{array}$$

and define the typical set $T_{n}\left(P_{Y}\right)$ as

(52) $$\begin{array}{c} T_{n}\left(P_{Y}\right)=\{y^{n}:|{\hat{P}}_{y^{n}}\left(y\right)-P_{Y}\left(y\right)\left|\leq \theta_{n}P_{Y}\left(y\right)\forall y\in Y\right\}. \end{array}$$

Using the Chernoff bound, we conclude that when *n* is sufficiently large,

(53) $$\begin{array}{c} P_{Y}^{n}\left(T_{n}\left(P_{Y}\right)\right)\geq 1-\frac{1-\varepsilon_{1}-\varepsilon_{2}}{4}. \end{array}$$

Now, define the following set

(54) $$\begin{array}{cc} C_{n} & :=B_{n}\cap D_{Y,n}\cap \left(X^{n}\times T_{n}\left(P_{Y}\right)\right). \end{array}$$

Then, combining (42), (49) and (53), we conclude that when *n* is sufficiently large,

(55) $$\begin{array}{c} P_{XY}^{n}\left(C_{n}\right)\geq 1-P_{XY}^{n}\left(B_{n}^{c}\right)-P_{XY}^{n}\left(D_{Y,n}^{c}\right)-P_{Y}^{n}\left(T_{n}^{c}\left(P_{Y}\right)\right)\geq \frac{1-\varepsilon_{1}-\varepsilon_{2}}{2}. \end{array}$$

Let the truncated distribution $P_{{\tilde{X}}^{n}{\tilde{Y}}^{n}{\tilde{Z}}^{n}}$ be defined as

(56) $$\begin{array}{cc} P_{{\tilde{X}}^{n}{\tilde{Y}}^{n}{\tilde{Z}}^{n}}\left(x^{n},y^{n},z^{n}\right) & :=\frac{P_{XY}^{n}\left(x^{n},y^{n}\right)1\left\{\left(x^{n},y^{n}\right)\in C_{n}\right\}}{P_{XY}^{n}\left(C_{n}\right)}P_{Z|Y}^{n}\left(z^{n}|y^{n}\right). \end{array}$$

Note that under our constructed truncated distribution $P_{{\tilde{X}}^{n}{\tilde{Y}}^{n}{\tilde{Z}}^{n}}$, the Markov chain ${\tilde{X}}^{n}-{\tilde{Y}}^{n}-{\tilde{Z}}^{n}$ holds.In other words, the Markovian property of the original source distribution $P_{XYZ}^{n}$ is retained for the truncated distribution $P_{{\tilde{X}}^{n}{\tilde{Y}}^{n}{\tilde{Z}}^{n}}$, which appears to be necessary to obtain a tight result if one wishes to use weak converse techniques. This is critical for our subsequent analyses.

Using the result in (55), we have that the marginal distribution $P_{{\tilde{X}}^{n}}$ satisfies that for any $x^{n}\in X^{N}$,

(57) $$\begin{array}{cc} P_{{\tilde{X}}^{n}}\left(x^{n}\right) & =\sum_{y^{n},z^{n}}P_{{\tilde{X}}^{n}{\tilde{Y}}^{n}{\tilde{Z}}^{n}}\left(x^{n},y^{n},z^{n}\right) \end{array}$$

(58) $$\begin{array}{cc} & \leq \frac{P_{X}^{n}\left(x^{n}\right)}{P_{XY}^{n}\left(C_{n}\right)}\leq \frac{2P_{X}^{n}\left(x^{n}\right)}{1-\varepsilon_{1}-\varepsilon_{2}}. \end{array}$$

Analogously to (58), we obtain that

(59) $$\begin{array}{cc} P_{{\tilde{Y}}^{n}}\left(y^{n}\right) & \leq \frac{2P_{Y}^{n}\left(y^{n}\right)}{1-\varepsilon_{1}-\varepsilon_{2}},\;\forall \;y^{n}\in Y^{n}, \end{array}$$

(60) $$\begin{array}{cc} P_{{\tilde{Z}}^{n}}\left(z^{n}\right) & \leq \frac{2P_{Z}^{n}\left(z^{n}\right)}{1-\varepsilon_{1}-\varepsilon_{2}},\;\forall \;z^{n}\in Z^{n}. \end{array}$$

Finally, note that

(61) $$\begin{array}{cc} D(P_{{\tilde{X}}^{n}{\tilde{Y}}^{n}{\tilde{Z}}^{n}}∥P_{XYZ}^{n}) & =D(P_{{\tilde{X}}^{n}{\tilde{Y}}^{n}}∥P_{XY}^{n}) \end{array}$$

(62) $$\begin{array}{cc} & =\log\frac{1}{P_{XY}^{n}\left(C_{n}\right)} \end{array}$$

(63) $$\begin{array}{cc} & \leq \log\frac{2}{1-\varepsilon_{1}-\varepsilon_{2}}. \end{array}$$

### 4.4. Step 2: Analyses of the Error Exponents of Type-II Error Probabilities

#### 4.4.1. Type-II Error Probability $\beta_{2}$ at the Relay

Let ${\tilde{M}}_{1}$ and ${\tilde{M}}_{2}$ be the outputs of encoders $f_{1}$ and $f_{2}$ respectively when the tuple of source sequences $\left({\tilde{X}}^{n},{\tilde{Y}}^{n},{\tilde{Z}}^{n}\right)$ is distributed according to $P_{{\tilde{X}}^{n}{\tilde{Y}}^{n}{\tilde{Z}}^{n}}$ defined in (56). Thus, recalling the definitions in (10), (11) and (56), we find that the joint distribution of $\left({\tilde{X}}^{n},{\tilde{Y}}^{n},{\tilde{Z}}^{n},{\tilde{M}}_{1},{\tilde{M}}_{2}\right)$ is given by

(64) $$\begin{array}{c} P_{{\tilde{X}}^{n}{\tilde{Y}}^{n}{\tilde{Z}}^{n}{\tilde{M}}_{1}{\tilde{M}}_{2}}\left(x^{n},y^{n},z^{n},m_{1},m_{2}\right)=P_{{\tilde{X}}^{n}{\tilde{Y}}^{n}{\tilde{Z}}^{n}}\left(x^{n},y^{n},z^{n}\right)P_{M_{1}|X^{n}}\left(m_{1}|x^{n}\right)P_{M_{2}|Y^{n}M_{1}}\left(m_{2}|y^{n},m_{1}\right). \end{array}$$

Let $P_{{\tilde{M}}_{1}{\tilde{Y}}^{n}}$ be induced by $P_{{\tilde{X}}^{n}{\tilde{Y}}^{n}{\tilde{Z}}^{n}{\tilde{M}}_{1}{\tilde{M}}_{2}}$. Combining (8) and (56), we conclude that

(65) $$\begin{array}{cc} P_{{\tilde{M}}_{1}{\tilde{Y}}^{n}}\left(A_{Y,n}\right) & =\sum_{\begin{array}{c} x^{n},y^{n},z^{n},m_{1},m_{2}:\\ g_{1}\left(m_{1},y^{n}\right)=H_{0} \end{array}}P_{{\tilde{X}}^{n}{\tilde{Y}}^{n}{\tilde{Z}}^{n}{\tilde{M}}_{1}{\tilde{M}}_{2}}\left(x^{n},y^{n},z^{n},m_{1},m_{2}\right) \end{array}$$

(66) $$\begin{array}{cc} & =\sum_{x^{n},y^{n}:g_{1}\left(f_{1}\left(x^{n}\right),y^{n}\right)=H_{0}}\frac{P_{XY}^{n}\left(x^{n},y^{n}\right)1\left\{\left(x^{n},y^{n}\right)\in C_{n}\right\}}{P_{XY}^{n}\left(C_{n}\right)} \end{array}$$

(67) $$\begin{array}{cc} & =\sum_{x^{n},y^{n}}\frac{P_{XY}^{n}\left(x^{n},y^{n}\right)1\left\{\left(x^{n},y^{n}\right)\in C_{n}\right\}}{P_{XY}^{n}\left(C_{n}\right)} \end{array}$$

(68) $$\begin{array}{cc} & =1. \end{array}$$

where (67) follows from the definition of $D_{Y,n}$ in (40) and the fact that $D_{Y,n}\subseteq C_{n}$.

Thus, using the data processing inequality for the relative entropy and the definition of $\beta_{2}$ in (15), we obtain that

(69) $$\begin{array}{cc} D(P_{{\tilde{M}}_{1}{\tilde{Y}}^{n}}∥P_{M_{1}}P_{Y}^{n}) & \geq D_{b}\left(P_{{\tilde{M}}_{1}{\tilde{Y}}^{n}}\left(A_{Y,n}\right)∥P_{M_{1}}P_{Y}^{n}\left(A_{Y,n}\right)\right) \end{array}$$

(70) $$\begin{array}{cc} & =-\log\left(P_{M_{1}}P_{Y}^{n}\left(A_{Y,n}\right)\right) \end{array}$$

(71) $$\begin{array}{cc} & =-\log\beta_{2}. \end{array}$$

Furthermore, recalling that $M_{1}$ denotes the output of encoder $f_{1}$ when $\left(X^{n},Y^{n},Z^{n}\right)∼P_{XYZ}^{n}$ and ${\tilde{M}}_{1}$ denotes the output of encoder $f_{1}$ when $\left(X^{n},Y^{n},Z^{n}\right)∼P_{{\tilde{X}}^{n}{\tilde{Y}}^{n}{\tilde{Z}}^{n}}$, and using the result in (58), we conclude that

(72) $$\begin{array}{cc} P_{{\tilde{M}}_{1}}\left(m_{1}\right) & =\sum_{x^{n},y^{n},z^{n}:f_{1}\left(x^{n}\right)=m_{1}}P_{{\tilde{X}}^{n}{\tilde{Y}}^{n}{\tilde{Z}}^{n}}\left(x^{n},y^{n},z^{n}\right) \end{array}$$

(73) $$\begin{array}{cc} & =\sum_{x^{n}:f_{1}\left(x^{n}\right)=m_{1}}P_{{\tilde{X}}^{n}}\left(x^{n}\right) \end{array}$$

(74) $$\begin{array}{cc} & \leq \sum_{x^{n}:f_{1}\left(x^{n}\right)=m_{1}}\frac{2P_{X}^{n}\left(x^{n}\right)}{1-\varepsilon_{1}-\varepsilon_{2}} \end{array}$$

(75) $$\begin{array}{cc} & \leq \frac{2P_{M_{1}}\left(m_{1}\right)}{1-\varepsilon_{1}-\varepsilon_{2}}, \end{array}$$

for any $m_{1}\in M_{1}$. Thus, combining (59), (71) and (75), we have

(76) $$\begin{array}{cc} -\log\beta_{2} & \leq D(P_{{\tilde{M}}_{1}{\tilde{Y}}^{n}}∥P_{M_{1}}P_{Y}^{n}) \end{array}$$

(77) $$\begin{array}{cc} & =D\left(P_{{\tilde{M}}_{1}{\tilde{Y}}^{n}}∥P_{{\tilde{M}}_{1}}P_{{\tilde{Y}}^{n}}\right)+E_{P_{{\tilde{M}}_{1}{\tilde{Y}}^{n}}}\left[\log\frac{P_{{\tilde{M}}_{1}}\left({\tilde{M}}_{1}\right)P_{{\tilde{Y}}^{n}}\left({\tilde{Y}}^{n}\right)}{P_{M_{1}}\left({\tilde{M}}_{1}\right)P_{Y}^{n}\left({\tilde{Y}}^{n}\right)}\right] \end{array}$$

(78) $$\begin{array}{cc} & \leq D\left(P_{{\tilde{M}}_{1}{\tilde{Y}}^{n}}∥P_{{\tilde{M}}_{1}}P_{{\tilde{Y}}^{n}}\right)+E_{P_{{\tilde{M}}_{1}{\tilde{Y}}^{n}}}\left[\log\frac{\frac{2P_{M_{1}}\left({\tilde{M}}_{1}\right)}{1-\varepsilon_{1}-\varepsilon_{2}}\frac{2P_{Y}^{n}\left({\tilde{Y}}^{n}\right)}{1-\varepsilon_{1}-\varepsilon_{2}}}{P_{M_{1}}\left({\tilde{M}}_{1}\right)P_{Y}^{n}\left({\tilde{Y}}^{n}\right)}\right] \end{array}$$

(79) $$\begin{array}{cc} & =I\left(\tilde{M};{\tilde{Y}}^{n}\right)+2\log\frac{2}{1-\varepsilon_{1}-\varepsilon_{2}}. \end{array}$$

#### 4.4.2. Type-II Error Probability $\eta_{2}$ at the Receiver

In this subsection, we analyze the error exponent of the type-II error probability at the receiver. For this purpose, we make use of the method introduced in [12] based on reverse hypercontractivity. We define the following additional notation:

- Give $P_{YZ}\in P\left(Y\times Z\right)$, define
  (80) $$\begin{array}{c} \alpha :=\max_{y,z}\frac{P_{Z|Y}\left(z|y\right)}{P_{Z}\left(z\right)}\in \left(1,\infty \right). \end{array}$$
  In the subsequent analysis, we only consider the case when α>1. When α=1, choosing $t=\frac{1}{\sqrt{n}}$ instead of the choice in (101), we can obtain a similar upper bound for $-\log\eta_{2}$ as in (102), where the only difference is that $\Psi (n,\varepsilon_{1},\varepsilon_{2})$ should be replaced by another term scaling in order $\Theta (\sqrt{n})$.
- Given any $\left(\varepsilon_{1},\varepsilon_{2}\right)\in {\left(0,1\right)}^{2}$ such that $\varepsilon_{1}+\varepsilon_{2}<1$, let
  (81) $$\begin{array}{c} \Psi \left(n,\varepsilon_{1},\varepsilon_{2}\right):=2\sqrt{n\left(\alpha -1\right)\log\frac{1+3\varepsilon_{2}-\varepsilon_{1}}{1-\varepsilon_{1}-\varepsilon_{2}}}. \end{array}$$
- Give any $m_{2}\in M_{2}$ and $z^{n}\in Z^{n}$, let
  (82) $$\begin{array}{c} h\left(m_{2},z^{n}\right):=1\left\{z^{n}\in G\left(m_{2}\right)\right\}. \end{array}$$
- Two operators in ([12] (Equations (25), (26), (29)))
  (83) $$\begin{array}{cc} \Lambda_{\alpha ,t} & ={\left(\exp\left(-t\right)+\alpha \left(1-\exp\left(-t\right)\right)P_{Z}\right)}^{⊗n}, \end{array}$$
  (84) $$\begin{array}{cc} T_{y^{n},t} & =\prod_{i=1}^{n}\left(\exp\left(-t\right)+\left(1-\exp\left(-t\right)\right)P_{Z|y_{i}}\right). \end{array}$$

Note that in (84), we use the convenient notation $P_{Z|y}\left(z\right)=P_{Z|Y}\left(z|y\right)$. The two operators in (83) and (84) will be used to lower bound $D(P_{{\tilde{Z}}^{n}{\tilde{M}}_{2}}∥P_{Z}^{n}{\bar{P}}_{M_{2}})$ via a variational formula of the relative entropy (cf. ([12] (Section 4))).

Let $P_{{\tilde{Z}}^{n}{\tilde{M}}_{2}}$, $P_{{\tilde{Z}}^{n}|{\tilde{M}}_{2}}$, $P_{{\tilde{Z}}^{n}|{\tilde{Y}}^{n}}$ be induced by the joint distribution $P_{{\tilde{X}}^{n}{\tilde{Y}}^{n}{\tilde{Z}}^{n}{\tilde{M}}_{1}{\tilde{M}}_{2}}$ in (64) and let ${\bar{P}}_{M_{2}}$ be induced by the joint distribution ${\bar{P}}_{X^{n}Y^{n}Z^{n}M_{1}M_{2}}$ in (13). Invoking the variational formula for the relative entropy ([22] (Equation (2.4.67))) and recalling the notation $P\left(f\right)=E_{P}\left[f\right]$, we have

(85) $$\begin{array}{cc} D(P_{{\tilde{Z}}^{n}{\tilde{M}}_{2}}∥P_{Z}^{n}{\bar{P}}_{M_{2}}) & \geq P_{{\tilde{Z}}^{n}{\tilde{M}}_{2}}\left(\log\Lambda_{\alpha ,t}h\left({\tilde{M}}_{2},{\tilde{Z}}^{n}\right)\right)-\log\left(\left(P_{Z}^{n}{\bar{P}}_{M_{2}}\right)\left(\Lambda_{\alpha ,t}h\left(M_{2},Z^{n}\right)\right)\right). \end{array}$$

Given any $m_{2}\in M_{2}$, similar to ([12] (Equations (18)–(21))), we obtain

$$\begin{array}{c} P_{Z}^{n}\left(\Lambda_{\alpha ,t}h\left(m_{2},Z^{n}\right)\right) \end{array}$$

(86) $$\begin{array}{c} =P_{Z}^{n}\left({\left(\exp\left(-t\right)+\alpha \left(1-\exp\left(-t\right)\right)P_{Z}\right)}^{⊗n}h\left(m_{2},Z^{n}\right)\right) \end{array}$$

(87) $$\begin{array}{c} ={\left(\exp(-t)+\alpha (1-\exp(-t))\right)}^{n}P_{Z}^{n}\left(h(m_{2},Z^{n})\right) \end{array}$$

(88) $$\begin{array}{c} \leq \exp\left(\left(\alpha -1\right)nt\right)P_{Z}^{n}\left(h(m_{2},Z^{n})\right). \end{array}$$

Thus, averaging over $m_{2}$ with distribution ${\bar{P}}_{M_{2}}$ on both sides of (88), we have

$$\begin{array}{c} \left(P_{Z}^{n}{\bar{P}}_{M_{2}}\right)\left(\Lambda_{\alpha ,t}h\left(M_{2},Z^{n}\right)\right) \end{array}$$

(89) $$\begin{array}{c} \leq \exp\left(\left(\alpha -1\right)nt\right)\left({\bar{P}}_{M_{2}}P_{Z}^{n}\right)\left(h(M_{2},Z^{n})\right) \end{array}$$

(90) $$\begin{array}{c} =\exp\left(\left(\alpha -1\right)nt\right)\eta_{2}, \end{array}$$

where (90) follows from the definition of $\eta_{2}$ in (17).

Furthermore, given any ${\tilde{m}}_{2}\in M_{2}$, we obtain

(91) $$\begin{array}{c} P_{{\tilde{Z}}^{n}|{\tilde{m}}_{2}}\left(\log\Lambda_{\alpha ,t}h\left({\tilde{m}}_{2},{\tilde{Z}}^{n}\right)\right) \end{array}$$

(92) $$\begin{array}{c} =\left(\sum_{{\tilde{y}}^{n}}P_{{\tilde{Z}}^{n}|{\tilde{y}}^{n}}P_{{\tilde{Y}}^{n}|{\tilde{M}}_{2}}\left({\tilde{y}}^{n}|{\tilde{m}}_{2}\right)\right)\left(\log\Lambda_{\alpha ,t}h\left({\tilde{m}}_{2},{\tilde{Z}}^{n}\right)\right) \end{array}$$

(93) $$\begin{array}{c} =\sum_{{\tilde{y}}^{n}}P_{{\tilde{Y}}^{n}|{\tilde{M}}_{2}}\left({\tilde{y}}^{n}|{\tilde{m}}_{2}\right)P_{{\tilde{Z}}^{n}|{\tilde{y}}^{n}}\left(\log\Lambda_{\alpha ,t}h\left({\tilde{m}}_{2},{\tilde{Z}}^{n}\right)\right) \end{array}$$

(94) $$\begin{array}{c} \geq \sum_{{\tilde{y}}^{n}}P_{{\tilde{Y}}^{n}|{\tilde{M}}_{2}}\left({\tilde{y}}^{n}|{\tilde{m}}_{2}\right)P_{{\tilde{Z}}^{n}|{\tilde{y}}^{n}}\left(\log T_{y^{n},t}h\left({\tilde{m}}_{2},{\tilde{Z}}^{n}\right)\right) \end{array}$$

(95) $$\begin{array}{c} \geq \sum_{{\tilde{y}}^{n}}P_{{\tilde{Y}}^{n}|{\tilde{M}}_{2}}\left({\tilde{y}}^{n}|{\tilde{m}}_{2}\right)\left(1+\frac{1}{t}\right)\log P_{{\tilde{Z}}^{n}|{\tilde{y}}^{n}}\left(h({\tilde{m}}_{2},{\tilde{Z}}^{n})\right) \end{array}$$

(96) $$\begin{array}{c} =\left(1+\frac{1}{t}\right)\left(\sum_{{\tilde{y}}^{n}}P_{{\tilde{Y}}^{n}|{\tilde{M}}_{2}}\left({\tilde{y}}^{n}|{\tilde{m}}_{2}\right)\log P_{{\tilde{Z}}^{n}|{\tilde{y}}^{n}}\left(G\left({\tilde{m}}_{2}\right)\right)\right). \end{array}$$

where (94) follows from ([12] (Lemma 4)) and (95) follows similarly to ([12] (Equations (14)–(17))).

Thus, averaging on both sides of (96) over ${\tilde{m}}_{2}$ with distribution $P_{{\tilde{M}}_{2}}$ and using the definition of the joint distribution $P_{{\tilde{X}}^{n}{\tilde{Y}}^{n}{\tilde{Z}}^{n}{\tilde{M}}_{1}{\tilde{M}}_{2}}$ in (64), we obtain that

$$\begin{array}{c} P_{{\tilde{Z}}^{n}{\tilde{M}}_{2}}\left(\log\Lambda_{\alpha ,t}h\left({\tilde{M}}_{2},{\tilde{Z}}^{n}\right)\right) \end{array}$$

(97) $$\begin{array}{c} \geq \left(1+\frac{1}{t}\right)\left(\sum_{{\tilde{y}}^{n},{\tilde{m}}_{2}}P_{{\tilde{Y}}^{n}{\tilde{M}}_{2}}\left({\tilde{y}}^{n},{\tilde{m}}_{2}\right)\log P_{{\tilde{Z}}^{n}|{\tilde{y}}^{n}}\left(G\left({\tilde{m}}_{2}\right)\right)\right)\\ =\left(1+\frac{1}{t}\right)\sum_{{\tilde{x}}^{n},{\tilde{y}}^{n},{\tilde{m}}_{1},{\tilde{m}}_{2}}(P_{{\tilde{X}}^{n}{\tilde{Y}}^{n}}\left({\tilde{x}}^{n},{\tilde{y}}^{n}\right)1\left\{{\tilde{m}}_{1}=f_{1}\left({\tilde{x}}^{n}\right),{\tilde{m}}_{2}=f_{2}\left({\tilde{m}}_{1},{\tilde{y}}^{n}\right)\right\} \end{array}$$

(98) $$\begin{array}{c} \;\;\;\;\;\;\times \log\left(\sum_{{\tilde{z}}^{n}:g_{2}\left({\tilde{z}}^{n},{\tilde{m}}_{2}\right)=H_{0}}P_{Z|Y}^{n}\left({\tilde{z}}^{n}|{\tilde{y}}^{n}\right)\right)) \end{array}$$

(99) $$\begin{array}{c} =\left(1+\frac{1}{t}\right)\left(\sum_{\begin{array}{c} {\tilde{x}}^{n},{\tilde{y}}^{n} \end{array}}\frac{P_{XY}^{n}\left({\tilde{x}}^{n},{\tilde{y}}^{n}\right)1\left\{\left({\tilde{x}}^{n},{\tilde{y}}^{n}\right)\in C_{n}\right\}}{P_{XY}^{n}\left(C_{n}\right)}\log P_{Z|Y}^{n}\left(G\left({\tilde{x}}^{n},{\tilde{y}}^{n}\right)|{\tilde{y}}^{n}\right)\right) \end{array}$$

(100) $$\begin{array}{c} \geq \left(1+\frac{1}{t}\right)\log\frac{1-\varepsilon_{1}-\varepsilon_{2}}{1+3\varepsilon_{2}-\varepsilon_{1}}, \end{array}$$

where (100) follows from the definitions of $B_{n}$ in (45) and $C_{n}$ in (54).

Therefore, combining (85), (90) and (100) and choosing

(101) $$\begin{array}{c} t=\sqrt{\frac{1}{n(\alpha -1)}\log\frac{1+3\varepsilon_{2}-\varepsilon_{1}}{1-\varepsilon_{1}-\varepsilon_{2}}}, \end{array}$$

via simple algebra, we obtain that

(102) $$\begin{array}{cc} -\log\eta_{2} & \leq D\left(P_{{\tilde{Z}}^{n}{\tilde{M}}_{2}}∥P_{Z^{n}}{\bar{P}}_{M_{2}}\right)+\Psi \left(n,\varepsilon_{1},\varepsilon_{2}\right)-\log\frac{1-\varepsilon_{1}-\varepsilon_{2}}{1+3\varepsilon_{2}-\varepsilon_{1}}. \end{array}$$

In the following, we further upper bound $D(P_{{\tilde{Z}}^{n}{\tilde{M}}_{2}}∥P_{Z^{n}}{\bar{P}}_{M_{2}})$. For this purpose, define the following distribution

(103) $$\begin{array}{cc} {\bar{P}}_{{\tilde{M}}_{2}}\left(m_{2}\right) & :=\sum_{y^{n},m_{1}}P_{{\tilde{M}}_{1}}\left(m_{1}\right)P_{{\tilde{Y}}^{n}}\left(y^{n}\right)1\left\{m_{2}=f_{2}\left(m_{1},y^{n}\right)\right\}. \end{array}$$

Combining the results in (59) and (75), and recalling that ${\bar{P}}_{M_{2}}$ is induced by joint distribution ${\bar{P}}_{X^{n}Y^{n}Z^{n}M_{1}M_{2}}$ in (13), for any $m_{2}\in M_{2}$, we have

(104) $$\begin{array}{cc} {\bar{P}}_{{\tilde{M}}_{2}}\left(m_{2}\right) & \leq {\left(\frac{2}{1-\varepsilon_{1}-\varepsilon_{2}}\right)}^{2}\left(\sum_{y^{n},m_{1}}P_{M_{1}}\left(m_{1}\right)P_{Y}^{n}\left(y^{n}\right)1\left\{m_{2}=f_{2}\left(f_{1}\left(x^{n}\right),y^{n}\right)\right\}\right) \end{array}$$

(105) $$\begin{array}{cc} & =\frac{4{\bar{P}}_{M_{2}}\left(m_{2}\right)}{{\left(1-\varepsilon_{1}-\varepsilon_{2}\right)}^{2}}. \end{array}$$

Thus, combining (60) and (105), we have

$$\begin{array}{c} D(P_{{\tilde{Z}}^{n}{\tilde{M}}_{2}}∥P_{Z}^{n}{\bar{P}}_{M_{2}}) \end{array}$$

(106) $$\begin{array}{c} =D\left(P_{{\tilde{Z}}^{n}{\tilde{M}}_{2}}∥P_{{\tilde{Z}}^{n}}{\bar{P}}_{{\tilde{M}}_{2}}\right)+E_{P_{{\tilde{Z}}^{n}{\tilde{M}}_{2}}}\left[\log\frac{P_{{\tilde{Z}}^{n}}\left({\tilde{Z}}^{n}\right){\bar{P}}_{{\tilde{M}}_{2}}\left({\tilde{M}}_{2}\right)}{P_{Z}^{n}\left({\tilde{Z}}^{n}\right){\bar{P}}_{M_{2}}\left({\tilde{M}}_{2}\right)}\right] \end{array}$$

(107) $$\begin{array}{c} \leq D\left(P_{{\tilde{Z}}^{n}{\tilde{M}}_{2}}∥P_{{\tilde{Z}}^{n}}{\bar{P}}_{{\tilde{M}}_{2}}\right)+E_{P_{{\tilde{Z}}^{n}{\tilde{M}}_{2}}}\left[\log\frac{\frac{2P_{Z}^{n}\left({\tilde{Z}}^{n}\right)}{1-\varepsilon_{1}-\varepsilon_{2}}\frac{4{\bar{P}}_{M_{2}}\left({\tilde{M}}_{2}\right)}{{\left(1-\varepsilon_{1}-\varepsilon_{2}\right)}^{2}}}{P_{Z}^{n}\left({\tilde{Z}}^{n}\right){\bar{P}}_{M_{2}}\left({\tilde{M}}_{2}\right)}\right] \end{array}$$

(108) $$\begin{array}{c} =D\left(P_{{\tilde{Z}}^{n}{\tilde{M}}_{2}}∥P_{{\tilde{Z}}^{n}}{\bar{P}}_{{\tilde{M}}_{2}}\right)+3\log\frac{2}{1-\varepsilon_{1}-\varepsilon_{2}}. \end{array}$$

Therefore, combining (102) and (108), we have

(109) $$\begin{array}{cc} -\log\eta_{2} & \leq D\left(P_{{\tilde{Z}}^{n}{\tilde{M}}_{2}}∥P_{{\tilde{Z}}^{n}}{\bar{P}}_{{\tilde{M}}_{2}}\right)+\Psi \left(n,\varepsilon_{1},\varepsilon_{2}\right)-\log\frac{1-\varepsilon_{1}-\varepsilon_{2}}{1+3\varepsilon_{2}-\varepsilon_{1}}-3\log\frac{1-\varepsilon_{1}-\varepsilon_{2}}{2}. \end{array}$$

### 4.5. Step 3: Analyses of Communication Constraints and Single-Letterization Steps

For any $\left(n,N_{1},N_{2}\right)$-code, since ${\tilde{M}}_{i}\in M_{i}$ for i∈{1,2}, we have that

(110) $$\begin{array}{cc} \log N_{1} & \geq H\left({\tilde{M}}_{1}\right)\geq I\left({\tilde{M}}_{1};{\tilde{X}}^{n},{\tilde{Y}}^{n}\right), \end{array}$$

(111) $$\begin{array}{cc} \log N_{2} & \geq H\left({\tilde{M}}_{2}\right)\geq I\left({\tilde{M}}_{2};{\tilde{Y}}^{n}\right). \end{array}$$

Furthermore, from the problem setting (see (64)), we have

(112) $$\begin{array}{c} I({\tilde{M}}_{1};{\tilde{Y}}^{n}|{\tilde{X}}^{n})=0, \end{array}$$

For subsequent analyses, given any $\left(b,c,d,\gamma \right)\in R_{+}^{4}$, define

(113) $$\begin{array}{cc} R_{b,c,d,\gamma }^{\left(n\right)} & :=-I\left({\tilde{M}}_{1};{\tilde{Y}}^{n}\right)+bI\left({\tilde{M}}_{1};{\tilde{X}}^{n},{\tilde{Y}}^{n}\right)-cD\left(P_{{\tilde{Z}}^{n}{\tilde{M}}_{2}}∥P_{{\tilde{Z}}^{n}}{\bar{P}}_{{\tilde{M}}_{2}}\right)+dI\left({\tilde{M}}_{2};{\tilde{Y}}^{n}\right)+\gamma I\left({\tilde{M}}_{1};{\tilde{Y}}^{n}|{\tilde{X}}^{n}\right)\\ & \;+\left(b+d+\gamma \right)D\left(P_{{\tilde{X}}^{n}{\tilde{Y}}^{n}}∥P_{X^{n}Y^{n}}\right). \end{array}$$

Combining the results in (63), (79), (109) to (112), for any $\gamma \in R_{+}$, we obtain

(114) $$\begin{array}{cc} & \log\beta_{2}+b\log N_{1}+c\log\eta_{2}+d\log N_{2}+c\Psi \left(n,\varepsilon_{1},\varepsilon_{2}\right)\\ & \geq R_{b,c,d,\gamma }^{\left(n\right)}+\log\frac{1-\varepsilon_{1}-\varepsilon_{2}}{1+3\varepsilon_{2}-\varepsilon_{1}}+\left(b+d+\gamma +5\right)\log\frac{1-\varepsilon_{1}-\varepsilon_{2}}{2}. \end{array}$$

The proof of Theorem 2 is complete by the two following lemmas which provide a single-letterized lower bound for $R_{b,c,d,\gamma }^{\left(n\right)}$ and relate the derived lower bound to $R_{b,c,d}$. For this purpose, recalling the definition of $\theta_{n}$ in (51), we define the following set of joint distributions

(115) $$\begin{array}{cc} Q_{1} & :=\{Q_{XYZUV}\in P\left(X\times Y\times Z\times U\times V\right):\\ & \;Q_{Z|Y}=P_{Z|Y},X-Y-Z,\;V-Y-Z,\\ & \;|Q_{Y}\left(y\right)-P_{Y}\left(y\right)|\leq \theta_{n}P_{Y}\left(y\right),\forall y\in Y\}. \end{array}$$

Given $Q_{XYZUV}\in Q_{1}$, define

(116) $$\begin{array}{cc} \Delta_{b,d,\gamma }\left(Q_{XYZUV}\right) & :=\left(b+\gamma \right)D\left(Q_{XY}∥P_{XY}\right)+dD\left(Q_{Y}∥P_{Y}\right)+\gamma I_{Q}\left(U;Y|X\right). \end{array}$$

Recall the definition of $R_{b,c,d}\left(Q_{XYZUV}\right)$ in (28). Define

(117) $$\begin{array}{cc} R_{b,c,d,\gamma } & :=\min_{Q_{XYZUV}\in Q_{1}}\left(R_{b,c,d}\left(Q_{XYZUV}\right)+\Delta_{b,d,\gamma }\left(Q_{XYZUV}\right)\right). \end{array}$$

The following lemma presents a single-letterized lower bound for $R_{b,c,d,\gamma }^{\left(n\right)}$.

**Lemma** **1.** *For any $\left(b,c,d,\gamma \right)\in R_{+}^{4}$,*

(118) $$\begin{array}{c} R_{b,c,d,\gamma }^{\left(n\right)}\geq nR_{b,c,d,\gamma }. \end{array}$$

The proof of Lemma 1 is inspired by ([13] (Prop. 2)) and provided in Appendix B.

Combining the results in (114) and Lemma 1, we obtain the desired result and this completes the proof of Theorem 2.

**Lemma** **2.** *Choosing $\gamma =\sqrt{n}$, we have*

(119) $$\begin{array}{c} nR_{b,c,d,\gamma }+\log\frac{1-\varepsilon_{1}-\varepsilon_{2}}{1+3\varepsilon_{2}-\varepsilon_{1}}+\left(b+d+\gamma +5\right)\log\frac{1-\varepsilon_{1}-\varepsilon_{2}}{2}\geq nR_{b,c,d}+\Theta \left(n^{3/4}\log n\right). \end{array}$$

The proof of Lemma 2 is inspired by ([19] (Lemma C.2)) and provided in Appendix C.

## 5. Discussion and Future Work

We strengthened the result in ([11] (Corollary 1)) by deriving a strong converse theorem for hypothesis testing against independence over a two-hop network with communication constraints (see Figure 1). In our proof, we combined two recently proposed strong converse techniques [12,13]. The apparent necessity of doing so comes from the Markovian requirement in the source distribution (recall (1)) and is reflected in the construction of a truncated distribution in (56) to ensure the Markovian structure of the source sequences is preserved. Subsequently, due to this constraint, the application the strong converse technique by Tyagi and Watanabe in [13] was only amenable in analyzing the type-II error exponent at the relay. On the other hand, to analyze the type-II error exponent at the receiver, we need to carefully adapt the strong converse technique based on reverse hypercontractivity by Liu, van Handel and Verdú in [12]. Furthermore, to complete the proof, we carefully combine the single-letterization techniques in [12,13].

Another important take-home message is the techniques (or a subset of the techniques) used in this paper can be applied to strengthen the results of other multiterminal hypothesis testing against independence problems. If the source distribution has no Markov structure, it is usually the case that one can directly apply the technique by Tyagi and Watanabe [13] to obtain strong converse theorems. Such examples include [7,8,9]. On the other hand, if the source sequences admit Markovian structure, then it appears necessary to combine techniques in [12,13] to obtain strong converse theorems, just as it was done in this paper.

Finally, we discuss some avenues for future research. In this paper, we only derived the strong converse but not a second-order converse result as was done in ([12] (Section 4.4)) for the problem of hypothesis testing against independence with a communication constraint [1]. Thus, in the future, one may refine the proof in the current paper by deriving second-order converse or exact second-order asymptotics. Furthermore, one may also consider deriving strong converse theorems or simplifying existing strong converse proofs for hypothesis testing problems with both communication and privacy constraints such as that in [23] by using the techniques in the current paper. It is also interesting to explore whether current techniques can be applied to obtain strong converse theorems for hypothesis testing with zero-rate compression problems [3].

## Appendix A. Achievability Proof of Proposition 1

Fix any joint distribution $Q_{XYZU_{1}U_{2}V}\in Q_{2}$. Let $\left(f_{1}^{\prime },g_{1}^{\prime }\right)$ be an encoder-decoder pair with rate $R_{1}=I_{Q}\left(U_{1};X\right)$ for the hypothesis testing with communication constraint problem [1] (i.e., no receiver in Figure 1) such that the type-II error probability decays exponentially fast at speed no smaller than $E_{1}=I_{Q}\left(U_{1};Y\right)$ and the type-I error probability is vanishing, i.e., $\log N_{1}^{\prime }\leq nR_{1}$, $\beta_{2}^{\prime }\leq \exp\left(-nE_{1}\right)$ and $\beta_{1}^{\prime }\leq \varepsilon_{1}^{\prime }$ for any $\varepsilon_{1}^{\prime }>0$. Furthermore, let $\left(f_{1}^{\prime \prime },f_{2}^{\prime \prime },g_{1}^{\prime \prime },g_{2}^{\prime \prime }\right)$ be a tuple of encoders and decoders with rates $\left(R_{1},R_{2}\right)=\left(I_{Q}\left(U_{2};X\right),I_{Q}\left(V;Y\right)\right)$ for the problem in Figure 1 such that the type-II error probability at the receiver decays exponentially fast at speed no smaller $E_{2}=I_{Q}\left(V;Z\right)$ and type-I error probability at the receiver is vanishing, i.e., $\log N_{1}^{\prime \prime }\leq nR_{1}$, $\log N_{2}^{\prime \prime }\leq nR_{2}$, $\eta_{2}^{\prime \prime }\leq \exp\left(-nE_{2}\right)$ and $\eta_{1}^{\prime \prime }\leq \varepsilon_{2}^{\prime }$ for any $\varepsilon_{2}^{\prime }>0$. Such tuples of encoders and decoders exist as proved in [1,11]. Furthermore, let $A_{1}^{\prime }\subseteq X^{n}\times Y^{n}$ be the acceptance region associated with $\left(f_{1}^{\prime },g_{1}^{\prime }\right)$ at the relay and let $A_{2}^{\prime }\subseteq X^{n}\times Y^{n}\times Z^{n}$ be the acceptance region associated with $\left(f_{1}^{\prime \prime },f_{2}^{\prime \prime },g_{1}^{\prime \prime },g_{2}^{\prime \prime }\right)$ at the receiver.

Now, let us partition the source space $X^{n}$ into two disjoint sets $X_{1}^{n}$ and $X_{2}^{n}$ such that $X_{1}^{n}\cup X_{2}^{n}=X^{n}$, $P_{X}^{n}\left(X_{1}^{n}\right)>1-\varepsilon_{1}$ and $P_{X}^{n}\left(X_{2}^{n}\right)>1-\varepsilon_{2}$. We construct an $\left(n,N_{1},N_{2}\right)$-code as follows. Given a source sequence $X^{n}$, if $X^{n}\in X_{1}^{n}$, then encoder $f_{1}^{\prime }$ is used; and if otherwise, the encoder $f_{1}^{\prime \prime }$ is used. Furthermore, an additional bit indicating whether $X^{n}\in X_{1}^{n}$ is also sent to the relay and further forwarded to the receiver by the relay. Given encoded index $M_{1}$, if $X^{n}\in X_{1}^{n}$, the relay uses decoder $g_{1}^{\prime }$ to make the decision; otherwise, if $X^{n}\in X_{2}^{n}$, the relay declares hypothesis $H_{1}$ to be true. Furthermore, in both cases, the relay transmits an index $M_{2}$ using encoder $f_{2}^{\prime \prime }$. Given the index $M_{2}$, if $X^{n}\in X_{1}^{n}$, the receiver declares hypothesis $H_{1}$ to be true; otherwise, the receiver uses decoder $g_{2}^{\prime \prime }$ to make the decision.

The performance of the constructed $\left(n,N_{1},N_{2}\right)$-code is as follows. In terms of rates, we have

(A1) $$\begin{array}{c} \log N_{1}\leq nR_{1}+1, \end{array}$$

(A2) $$\begin{array}{c} \log N_{2}\leq nR_{2}+1. \end{array}$$

The type-I error probability at the relay satisfies that

(A3) $$\begin{array}{cc} 1-\beta_{1} & =P_{XY}^{n}\left\{A_{1}^{\prime }\cap \left(X_{1}^{n}\times Y^{n}\right)\right\} \end{array}$$

(A4) $$\begin{array}{cc} & \geq P_{X}^{n}\left\{X_{1}^{n}\right\}-P_{XY}^{n}\left\{{\left(A_{1}^{\prime }\right)}^{c}\right\} \end{array}$$

(A5) $$\begin{array}{cc} & \geq 1-\varepsilon_{1}, \end{array}$$

where (A5) follows when *n* is sufficiently large and thus $\varepsilon_{1}^{\prime }$ can be made arbitrarily close to zero. Furthermore, the type-II error probability at the relay can be upper bounded as follows

(A6) $$\begin{array}{cc} \beta_{2} & =P_{X}^{n}P_{Y}^{n}\left\{A_{1}^{\prime }\cap \left(X_{1}^{n}\times Y^{n}\right)\right\} \end{array}$$

(A7) $$\begin{array}{cc} & \leq P_{X}^{n}P_{Y}^{n}\left\{A_{1}^{\prime }\right\} \end{array}$$

(A8) $$\begin{array}{cc} & =\beta_{2}^{\prime } \end{array}$$

(A9) $$\begin{array}{cc} & \leq \exp(-nE_{1}^{\prime }). \end{array}$$

Similarly, for *n* sufficiently large, the error probabilities at the receiver can be upper bounded as follows

(A10) $$\begin{array}{cc} \eta_{1} & =1-P_{XYZ}^{n}\left\{A_{2}^{\prime \prime }\cap \left(X_{2}^{n}\times Y^{n}\times Z^{n}\right)\right\} \end{array}$$

(A11) $$\begin{array}{cc} & \leq 1-P_{X}^{n}\left(X_{2}^{n}\right)+P_{XYZ}^{n}\left({\left(A_{2}^{\prime }\right)}^{c}\right) \end{array}$$

(A12) $$\begin{array}{cc} & \leq \varepsilon_{2}, \end{array}$$

and

(A13) $$\begin{array}{cc} \eta_{2} & =P_{X}^{n}P_{Y}^{n}P_{Z}^{n}\left\{A_{2}^{\prime \prime }\cap \left(X_{2}^{n}\times Y^{n}\times Z^{n}\right)\right\} \end{array}$$

(A14) $$\begin{array}{cc} & \leq P_{X}^{n}P_{Y}^{n}P_{Z}^{n}\left\{A_{2}^{\prime \prime }\right\} \end{array}$$

(A15) $$\begin{array}{cc} & \leq \exp(-nE_{2}^{\prime \prime }). \end{array}$$

The achievability proof of Proposition 1 is now complete.

## Appendix B. Proof of Lemma 1

Recall the definition of distribution ${\bar{P}}_{{\tilde{M}}_{2}}$ (see (103)). Noting that $P_{{\tilde{M}}_{2}}$ is the marginal distribution induced by $P_{{\tilde{X}}^{n}{\tilde{Y}}^{n}{\tilde{Z}}^{n}{\tilde{M}}_{1}{\tilde{M}}_{2}}$ (see (64)), we have that for any ${\tilde{m}}_{2}\in M_{2}$

(A16) $$\begin{array}{cc} P_{{\tilde{M}}_{2}}\left({\tilde{m}}_{2}\right) & =\sum_{y^{n},m_{1}}P_{{\tilde{Y}}^{n}{\tilde{M}}_{1}}\left(y^{n},m_{1}\right)1\left\{{\tilde{m}}_{2}=f_{2}\left(m_{1},y^{n}\right)\right\}. \end{array}$$

Thus, applying the data processing inequality for the relative entropy, we have that

(A17) $$\begin{array}{cc} I({\tilde{M}}_{1};{\tilde{Y}}^{n}) & =D(P_{{\tilde{Y}}^{n}{\tilde{M}}_{1}}∥P_{{\tilde{Y}}^{n}}P_{{\tilde{M}}_{1}}) \end{array}$$

(A18) $$\begin{array}{cc} & \geq D(P_{{\tilde{M}}_{2}}∥{\bar{P}}_{{\tilde{M}}_{2}}). \end{array}$$

Using (A18) and following similar steps to the proof of weak converse in ([11] (Equationation (186))), we obtain

(A19) $$\begin{array}{cc} D(P_{{\tilde{Z}}^{n}{\tilde{M}}_{2}}∥P_{{\tilde{Z}}^{n}}{\bar{P}}_{{\tilde{M}}_{2}}) & =I\left({\tilde{M}}_{2};{\tilde{Z}}^{n}\right)+D\left(P_{{\tilde{M}}_{2}}∥{\bar{P}}_{{\tilde{M}}_{2}}\right) \end{array}$$

(A20) $$\begin{array}{cc} & \leq I\left({\tilde{M}}_{2};{\tilde{Z}}^{n}\right)+I\left({\tilde{M}}_{1};{\tilde{Y}}^{n}\right). \end{array}$$

Using (A20) and the definition of $R_{b,c,d,\gamma }^{\left(n\right)}$ in (113), we have the following lower bound for $R_{b,c,d,\gamma }^{\left(n\right)}$

(A21) $$\begin{array}{cc} R_{b,c,d,\gamma }^{\left(n\right)} & \geq -I\left({\tilde{M}}_{1};{\tilde{Y}}^{n}\right)+b\left(D\left(P_{{\tilde{X}}^{n}{\tilde{Y}}^{n}}∥P_{X^{n}Y^{n}}\right)+H\left({\tilde{X}}^{n},{\tilde{Y}}^{n}\right)-H\left({\tilde{X}}^{n},{\tilde{Y}}^{n}|{\tilde{M}}_{1}\right)\right)\\ & -c\left(I\left({\tilde{M}}_{2};Z^{n}\right)+I\left({\tilde{M}}_{1};{\tilde{Y}}^{n}\right)\right)+d\left(D\left(P_{{\tilde{X}}^{n}{\tilde{Y}}^{n}}∥P_{X^{n}Y^{n}}\right)+H\left({\tilde{Y}}^{n}\right)-h\left({\tilde{Y}}^{n}|{\tilde{M}}_{2}\right)\right)\\ & +\gamma \left(D\left(P_{{\tilde{X}}^{n}{\tilde{Y}}^{n}}∥P_{X^{n}Y^{n}}\right)+H\left({\tilde{Y}}^{n}|{\tilde{X}}^{n}\right)-H\left({\tilde{Y}}^{n}|{\tilde{X}}^{n},{\tilde{M}}_{1}\right)\right). \end{array}$$

The rest of the proof concerns single-letterizing each term in (A21). For this purpose, for each j∈[n], we define two auxiliary random variables $U_{j}:=\left({\tilde{M}}_{1},{\tilde{X}}^{j-1},{\tilde{Y}}^{j-1}\right)$ and $V_{j}:=\left({\tilde{M}}_{2},{\tilde{Y}}^{j-1}\right)$ and let *J* be a random variable which is distributed uniformly over the set [n] and is independent of all other random variables.

Using standard single-letterization techniques as in [21], we obtain

(A22) $$\begin{array}{cc} I({\tilde{M}}_{1};{\tilde{Y}}^{n}) & =\sum_{j\in [n]}I\left({\tilde{M}}_{1};{\tilde{Y}}_{j}|{\tilde{Y}}^{j-1}\right) \end{array}$$

(A23) $$\begin{array}{cc} & \leq \sum_{j\in [n]}I\left({\tilde{M}}_{1},{\tilde{Y}}^{j-1};{\tilde{Y}}_{j}\right) \end{array}$$

(A24) $$\begin{array}{cc} & \leq \sum_{j\in [n]}I\left({\tilde{M}}_{1},{\tilde{X}}^{j-1},{\tilde{Y}}^{j-1};{\tilde{Y}}_{j}\right) \end{array}$$

(A25) $$\begin{array}{cc} & =nI(U_{J},J;{\tilde{Y}}_{J}), \end{array}$$

and

(A26) $$\begin{array}{cc} H({\tilde{X}}^{n},{\tilde{Y}}^{n}|{\tilde{M}}_{1}) & =nH({\tilde{X}}_{J},{\tilde{Y}}_{J}|U_{J},J). \end{array}$$

Furthermore, analogous to ([13] (Prop. 1)), we obtain that

(A27) $$\begin{array}{cc} H\left({\tilde{X}}^{n},{\tilde{Y}}^{n}\right)+D\left(P_{{\tilde{X}}^{n}{\tilde{Y}}^{n}}∥P_{XY}^{n}\right) & =\sum_{x^{n},y^{n}}P_{{\tilde{X}}^{n}{\tilde{Y}}^{n}}\left(x^{n},y^{n}\right)\log\frac{1}{P_{XY}^{n}\left(x^{n},y^{n}\right)} \end{array}$$

(A28) $$\begin{array}{cc} & =\sum_{x^{n},y^{n}}P_{{\tilde{X}}^{n}{\tilde{Y}}^{n}}\left(x^{n},y^{n}\right)\sum_{j\in [n]}\log\frac{1}{P_{XY}\left(x_{j},y_{j}\right)} \end{array}$$

(A29) $$\begin{array}{cc} & =\sum_{j\in [n]}P_{{\tilde{X}}_{j}{\tilde{Y}}_{j}}\left(x_{j},y_{j}\right)\log\frac{1}{P_{XY}\left(x_{j},y_{j}\right)} \end{array}$$

(A30) $$\begin{array}{cc} & =n\left(H\left({\tilde{X}}_{J},{\tilde{Y}}_{J}\right)+D\left(P_{{\tilde{X}}_{J}Y_{J}}∥P_{XY}\right)\right). \end{array}$$

Subsequently, we can single-letterize $I({\tilde{M}}_{2};{\tilde{Z}}^{n})$ as follows:

(A31) $$\begin{array}{cc} I({\tilde{M}}_{2};{\tilde{Z}}^{n}) & =\sum_{j\in [n]}I\left({\tilde{M}}_{2};{\tilde{Z}}_{j}|{\tilde{Z}}^{j-1}\right) \end{array}$$

(A32) $$\begin{array}{cc} & \leq \sum_{j\in [n]}I\left({\tilde{M}}_{2},{\tilde{Z}}^{j-1},{\tilde{Y}}^{j-1};{\tilde{Z}}_{j}\right) \end{array}$$

(A33) $$\begin{array}{cc} & =\sum_{j\in [n]}I\left({\tilde{M}}_{2},{\tilde{Y}}^{j-1};{\tilde{Z}}_{j}\right) \end{array}$$

(A34) $$\begin{array}{cc} & =nI(V_{J},J;{\tilde{Z}}_{J}), \end{array}$$

where (A33) follows from the Markov chain ${\tilde{Z}}^{j-1}-{\tilde{M}}_{2}{\tilde{Y}}^{j-1}-{\tilde{Z}}_{j}$ implied by the joint distribution of $\left({\tilde{X}}^{n},{\tilde{Y}}^{n},{\tilde{Z}}^{n},{\tilde{M}}_{1},{\tilde{M}}_{2}\right)$ in (64). Furthermore, using similar proof techniques to ([13] (Prop. 1)) and standard single-letterization techniques (e.g., in [4] or [21]), we obtain that

(A35) $$\begin{array}{cc} H\left({\tilde{Y}}^{n}|{\tilde{X}}^{n}\right)+D\left(P_{{\tilde{X}}^{n}{\tilde{Y}}^{n}}∥P_{XY}^{n}\right) & \geq n\left(H\left({\tilde{Y}}_{J}|{\tilde{X}}_{J}\right)+D\left(P_{{\tilde{X}}_{J}{\tilde{Y}}_{J}}∥P_{XY}\right)\right), \end{array}$$

(A36) $$\begin{array}{cc} H\left({\tilde{Y}}^{n}\right)+D\left(P_{{\tilde{X}}^{n}{\tilde{Y}}^{n}}∥P_{XY}^{n}\right) & \geq n\left(H\left({\tilde{Y}}_{J}\right)+D\left(P_{Y_{J}}∥P_{Y}\right)\right), \end{array}$$

(A37) $$\begin{array}{cc} H({\tilde{Y}}^{n}|{\tilde{M}}_{2}) & =nH({\tilde{Y}}_{J}|V_{J},J), \end{array}$$

(A38) $$\begin{array}{cc} H({\tilde{Y}}^{n}|{\tilde{M}}_{1},{\tilde{X}}^{n}) & \leq nH({\tilde{Y}}_{J}|X_{J},U_{J},J). \end{array}$$

Let $U:=(U_{J},J)$, $V:=(V_{J},J)$, $X^{\prime }:={\tilde{X}}_{J}$, $Y^{\prime }:={\tilde{Y}}_{J}$ and $Z^{\prime }:={\tilde{Z}}_{J}$. Using the joint distribution $P_{{\tilde{X}}^{n}{\tilde{Y}}^{n}{\tilde{Z}}^{n}{\tilde{M}}_{1}{\tilde{M}}_{2}}$ in (64), we conclude that the joint distribution of random variables $\left(X^{\prime },Y^{\prime },Z^{\prime },U,V\right)$, denoted by $Q_{X^{\prime }Y^{\prime }Z^{\prime }UV}$, belongs to the set $Q_{1}$ defined in (115). The proof of Lemma 1 is complete by combining (A21) to (A38) and noting that $I_{Q}\left(X^{\prime },Y^{\prime };U\right)\geq I_{Q}\left(X^{\prime };U\right)$.

## Appendix C. Proof of Lemma 2

Given any $\gamma \in R_{+}$, let $Q_{XYZUV}^{\left(\gamma \right)}$ achieve the minimum in (117). Recall the definition of $\theta_{n}$ in (51) and define a new alphabet $\tilde{V}:=V\cup \left\{v^{*}\right\}$. We then define a joint distribution $P_{Y\tilde{V}}^{\left(\gamma \right)}$ by specifying the following (conditional) marginal distributions

(A39) $$\begin{array}{cc} P_{\tilde{V}}^{\left(\gamma \right)}\left(v\right) & :=\frac{1}{1+\theta_{n}}Q_{V}^{\left(\gamma \right)}\left(v\right)1\left\{v\neq v^{*}\right\}+\frac{\theta_{n}}{1+\theta_{n}}1\left\{v=v^{*}\right\}, \end{array}$$

(A40) $$\begin{array}{cc} P_{Y|\tilde{V}}^{\left(\gamma \right)}\left(y|v\right) & :=Q_{Y|V}^{\left(\gamma \right)}\left(y|v\right)1\left\{v\neq v^{*}\right\}+\left(\frac{1+\theta_{n}}{\theta_{n}}P_{Y}\left(y\right)-\frac{1}{\theta_{n}}Q_{Y}^{\left(\gamma \right)}\left(y\right)\right)1\left\{v=v^{*}\right\}. \end{array}$$

Thus, the induced marginal distribution $P_{Y}^{\left(\gamma \right)}$ satisfies

(A41) $$\begin{array}{cc} P_{Y}^{\left(\gamma \right)}\left(y\right) & =\sum_{v\in \tilde{V}}P_{\tilde{V}}^{\left(\gamma \right)}\left(v\right)P_{Y|\tilde{V}}^{\left(\gamma \right)}\left(y|v\right) \end{array}$$

(A42) $$\begin{array}{cc} & =\left(\sum_{v\in V}\frac{1}{1+\theta_{n}}Q_{V}^{\left(\gamma \right)}\left(v\right)Q_{Y|V}^{\left(\gamma \right)}\left(y|v\right)\right)+\left(P_{Y}\left(y\right)-\frac{1}{1+\theta_{n}}Q_{Y}^{\left(\gamma \right)}\left(y\right)\right) \end{array}$$

(A43) $$\begin{array}{cc} & =P_{Y}\left(y\right). \end{array}$$

Furthermore, let $P_{\tilde{V}|Y}^{\left(\gamma \right)}$ be induced by $P_{Y\tilde{V}}^{\left(\gamma \right)}$ and define the following distribution

(A44) $$\begin{array}{c} P_{XYZU\tilde{V}}^{\left(\gamma \right)}=P_{XYZ}Q_{U|X}^{\left(\gamma \right)}P_{\tilde{V}|Y}^{\left(\gamma \right)}. \end{array}$$

Recall the definition of $R_{b,c,d}\left(\cdot \right)$ in (28). The following lemma lower bounds the difference between $R_{b,c,d}\left(Q_{XYZUV}^{\left(\gamma \right)}\right)$ and $R_{b,c,d}\left(P_{XYZU\tilde{V}}^{\left(\gamma \right)}\right)$ and is critical in the proof of Lemma 2.

**Lemma** **A1.**
*When $\gamma =\sqrt{n}$, we have*

(A45) $$\begin{array}{c} R_{b,c,d}\left(Q_{XYZUV}^{\left(\gamma \right)}\right)-R_{b,c,d}\left(P_{XYZU\tilde{V}}^{\left(\gamma \right)}\right)\geq \Theta \left(\frac{\log n}{n^{1/4}}\right). \end{array}$$

The proof of Lemma A1 is deferred to Appendix D.

Now, using the assumption that $Q_{XYZUV}^{\left(\gamma \right)}$ is a minimizer for $R_{b,c,d,\gamma }$ in (117), the fact that $\Delta_{b,d,\gamma }\left(Q_{XYZUV}^{\left(\gamma \right)}\right)\geq 0$ (see (116)) and the result in (A45), we conclude that when $\gamma =\sqrt{n}$,

(A46) $$\begin{array}{cc} R_{b,c,d,\gamma } & =R_{b,c,d}\left(Q_{XYZUV}^{\left(\gamma \right)}\right)+\Delta_{b,d,\gamma }\left(Q_{XYZUV}^{\left(\gamma \right)}\right) \end{array}$$

(A47) $$\begin{array}{cc} & \geq R_{b,c,d}\left(P_{XYZU\tilde{V}}^{\left(\gamma \right)}\right)+\Theta \left(\frac{\log n}{n^{1/4}}\right) \end{array}$$

(A48) $$\begin{array}{cc} & \geq R_{b,c,d}+\Theta \left(\frac{\log n}{n^{1/4}}\right), \end{array}$$

where (A48) follows from the definition of $R_{b,c,d}$ in (29) and the fact that $P_{XYZU\tilde{V}}^{\left(\gamma \right)}\in Q$ (see (24)).

The proof of Lemma 2 is complete by using (A48) and noting that when $\gamma =\sqrt{n}$,

(A49) $$\begin{array}{c} \log\frac{1-\varepsilon_{1}-\varepsilon_{2}}{1+3\varepsilon_{2}-\varepsilon_{1}}+\left(b+d+\gamma +5\right)\log\frac{1-\varepsilon_{1}-\varepsilon_{2}}{2}=\Theta \left(\sqrt{n}\right). \end{array}$$

## Appendix D. Proof of Lemma A1

In subsequent analyses, all distributions indicated by $P^{\left(\gamma \right)}$ are induced by $P_{XYZU\tilde{V}}^{\left(\gamma \right)}$. We have

(A50) $$\begin{array}{c} D\left(Q_{XYU}^{\left(\gamma \right)}∥P_{XYU}^{\left(\gamma \right)}\right)=D\left(Q_{XY}^{\left(\gamma \right)}∥P_{XY}^{\left(\gamma \right)}\right)+I_{Q^{\left(\gamma \right)}}\left(U;Y|X\right). \end{array}$$

Recalling the definitions of $R_{b,c,d}$ in (29) and $R_{b,c,d,\gamma }$ in (117), we conclude that for any $\gamma \in R_{+}$,

(A51) $$\begin{array}{c} R_{b,c,d,\gamma }\leq R_{b,c,d}\leq b\log|X|+d\log|Y|=:a^{\prime }. \end{array}$$

Using the definition of $\Delta_{b,d,\gamma }\left(Q_{XYZUV}\right)$ in (116) and recalling that $Q_{XYZUV}^{\left(\gamma \right)}$ is a minimizer for $R_{b,c,d,\gamma }$, we have

(A52) $$\begin{array}{cc} \gamma D(Q_{XYU}^{\left(\gamma \right)}∥P_{XYU}^{\left(\gamma \right)}) & \leq \Delta_{b,d,\gamma }\left(Q_{XYZUV}^{\left(\gamma \right)}\right) \end{array}$$

(A53) $$\begin{array}{cc} & =R_{b,c,d,\gamma }-R_{b,c,d}\left(Q_{XYZUV}^{\left(\gamma \right)}\right) \end{array}$$

(A54) $$\begin{array}{cc} & \leq a^{\prime }+\left(c+1\right)\log|Y|+c\log|Z|=:a. \end{array}$$

We can now upper bound $I_{P^{\gamma }}\left(\tilde{V};Y\right)$ as follows:

(A55) $$\begin{array}{cc} I_{P^{\left(\gamma \right)}}\left(\tilde{V};Y\right) & =D(P_{Y|\tilde{V}}^{\left(\gamma \right)}∥P_{Y}^{\left(\gamma \right)}|P_{\tilde{V}}^{\left(\gamma \right)}) \end{array}$$

(A56) $$\begin{array}{cc} & =D(P_{Y|\tilde{V}}^{\left(\gamma \right)}∥P_{Y}|P_{\tilde{V}}^{\left(\gamma \right)}) \end{array}$$

(A57) $$\begin{array}{cc} & =\frac{1}{1+\theta_{n}}D(Q_{Y|V}^{\left(\gamma \right)}∥P_{Y}\left|Q_{V}^{\left(\gamma \right)}\right)+\frac{\theta_{n}}{1+\theta_{n}}D\left(\frac{1+\theta_{n}}{\theta_{n}}P_{Y}-\frac{1}{\theta_{n}}Q_{Y}^{\left(\gamma \right)}∥P_{Y}\right) \end{array}$$

(A58) $$\begin{array}{cc} & =\frac{1}{1+\theta_{n}}\left(D(Q_{Y|V}^{\left(\gamma \right)}∥Q_{Y}^{\left(\gamma \right)}\left|Q_{V}^{\left(\gamma \right)}\right)+D\left(Q_{Y}^{\left(\gamma \right)}∥P_{Y}\right)\right)+\frac{\theta_{n}}{1+\theta_{n}}D\left(\frac{1+\theta_{n}}{\theta_{n}}P_{Y}-\frac{1}{\theta_{n}}Q_{Y}^{\left(\gamma \right)}∥P_{Y}\right) \end{array}$$

(A59) $$\begin{array}{cc} & \leq \frac{1}{1+\theta_{n}}I_{Q^{\left(\gamma \right)}}\left(V;Y\right)+\frac{1}{1+\theta_{n}}\frac{a}{\gamma }+\frac{\theta_{n}}{1+\theta_{n}}\log\mu , \end{array}$$

where (A56) follows from (A43), and (A59) follows from the result in (A54), the fact that $D\left(Q_{Y}^{\left(\gamma \right)}∥P_{Y}\right)\leq D\left(Q_{XYU}^{\left(\gamma \right)}∥P_{XYU}^{\left(\gamma \right)}\right)$ and the definition of μ in (50). Thus, when $\gamma =\sqrt{n}$, recalling the definition of $\theta_{n}$ in (51), we have

(A60) $$\begin{array}{cc} I_{Q^{\left(\gamma \right)}}\left(V;Y\right) & \geq I_{P^{\left(\gamma \right)}}\left(\tilde{V};Y\right)-\frac{a}{\gamma }-\theta_{n}\log\mu \end{array}$$

(A61) $$\begin{array}{cc} & =I_{P^{\left(\gamma \right)}}\left(\tilde{V};Y\right)+\Theta \left(\frac{1}{\sqrt{n}}\right). \end{array}$$

Similar to (A59), we obtain

$$\begin{array}{c} I_{P^{\left(\gamma \right)}}\left(\tilde{V};Z\right) \end{array}$$

(A62) $$\begin{array}{c} =D(P_{Z|\tilde{V}}^{\left(\gamma \right)}∥P_{Z}^{\left(\gamma \right)}|P_{\tilde{V}}^{\left(\gamma \right)}) \end{array}$$

(A63) $$\begin{array}{c} =D(P_{Z|\tilde{V}}^{\left(\gamma \right)}∥P_{Z}|P_{\tilde{V}}^{\left(\gamma \right)}) \end{array}$$

(A64) $$\begin{array}{c} =\frac{1}{1+\theta_{n}}D(Q_{Z|V}^{\left(\gamma \right)}∥P_{Z}\left|Q_{V}^{\left(\gamma \right)}\right)+\frac{\theta_{n}}{1+\theta_{n}}D\left(\frac{1+\theta_{n}}{\theta_{n}}P_{Z}-\frac{1}{\theta_{n}}Q_{Z}^{\left(\gamma \right)}∥P_{Z}\right) \end{array}$$

(A65) $$\begin{array}{c} =\frac{1}{1+\theta_{n}}\left(D(Q_{Z|V}^{\left(\gamma \right)}∥Q_{Z}^{\left(\gamma \right)}\left|Q_{V}^{\left(\gamma \right)}\right)+D\left(Q_{Z}^{\left(\gamma \right)}∥P_{Z}\right)\right)+\frac{\theta_{n}}{1+\theta_{n}}D\left(\frac{1+\theta_{n}}{\theta_{n}}P_{Z}-\frac{1}{\theta_{n}}Q_{Z}^{\left(\gamma \right)}∥P_{Z}\right) \end{array}$$

(A66) $$\begin{array}{c} \geq \frac{1}{1+\theta_{n}}I_{Q^{\left(\gamma \right)}}\left(V;Z\right), \end{array}$$

where (A64) follows since $Q^{\left(\gamma \right)}\in Q_{1}$ (see (115)) implies that $Q_{Z|Y}^{\left(\gamma \right)}=P_{Z|Y}$ and the Markov chains Z−Y−X and V−Y−Z holds and thus using (A39) to (A40), we have

(A67) $$\begin{array}{cc} P_{Z|\tilde{V}}^{\left(\gamma \right)}\left(z|v\right) & =\frac{\sum_{y}P_{Z|Y}\left(z|y\right)P_{\tilde{V}}\left(v\right)P_{Y|\tilde{V}}\left(y|v\right)}{P_{\tilde{V}}\left(v\right)} \end{array}$$

(A68) $$\begin{array}{cc} & =\frac{\sum_{y}Q_{Z|Y}^{\left(\gamma \right)}\left(z|y\right)Q_{V}^{\left(\gamma \right)}\left(v\right)Q_{Y|V}^{\left(\gamma \right)}\left(y|v\right)}{Q_{V}^{\left(\gamma \right)}\left(v\right)} \end{array}$$

(A69) $$\begin{array}{cc} & =Q_{Z|V}^{\left(\gamma \right)}\left(z|v\right), \end{array}$$

and

(A70) $$\begin{array}{cc} P_{Z|\tilde{V}}^{\left(\gamma \right)}\left(z|v^{*}\right) & =\frac{\sum_{y}P_{Z|Y}\left(z|y\right)P_{\tilde{V}}\left(v^{*}\right)P_{Y|\tilde{V}}\left(y|v^{*}\right)}{P_{\tilde{V}}\left(v^{*}\right)} \end{array}$$

(A71) $$\begin{array}{cc} & =\sum_{y}Q_{Z|Y}^{\left(\gamma \right)}\left(z|y\right)\left(\frac{1+\theta_{n}}{\theta_{n}}P_{Y}\left(y\right)-\frac{1}{\theta_{n}}Q_{Y}^{\left(\gamma \right)}\left(y\right)\right) \end{array}$$

(A72) $$\begin{array}{cc} & =\frac{1+\theta_{n}}{\theta_{n}}P_{Z}\left(z\right)-\frac{1}{\theta_{n}}Q_{Z}^{\left(\gamma \right)}\left(z\right), \end{array}$$

Therefore, we have

(A73) $$\begin{array}{cc} I_{Q^{\left(\gamma \right)}}\left(V;Z\right) & \leq \left(1+\theta_{n}\right)I_{P^{\left(\gamma \right)}}\left(\tilde{V};Z\right) \end{array}$$

(A74) $$\begin{array}{cc} & \leq I_{P^{\left(\gamma \right)}}\left(\tilde{V};Z\right)+\theta_{n}\log\left|Z\right| \end{array}$$

(A75) $$\begin{array}{cc} & =I_{P^{\left(\gamma \right)}}\left(\tilde{V};Z\right)+\Theta \left(\frac{1}{\sqrt{n}}\right). \end{array}$$

Let ∥P−Q∥ be the $\ell_{1}$ norm between *P* and *Q* regarded as vectors. Using Pinsker’s inequality, the result in (105), and the data processing inequality for the relative entropy [17], we obtain

(A76) $$\begin{array}{cc} ∥Q_{UX}^{\left(\gamma \right)}-P_{UX}^{\left(\gamma \right)}∥ & \leq \sqrt{2\log2\cdot D(Q_{UX}^{\left(\gamma \right)}∥P_{UX}^{\left(\gamma \right)})} \end{array}$$

(A77) $$\begin{array}{cc} & \leq \sqrt{2\log2\cdot D(Q_{XYU}^{\left(\gamma \right)}∥P_{XYU}^{\left(\gamma \right)})} \end{array}$$

(A78) $$\begin{array}{cc} & \leq \sqrt{\frac{2a\log2}{\gamma }}. \end{array}$$

From the support lemma ([21] (Appendix C)), we conclude that the cardinality of *U* can be upper bounded by a function depending only on |X|, |Y| and |Z| (these alphabets are all finite). Thus, when $\gamma =\sqrt{n}$, invoking ([4] (Lemma 2.2.7)), we have

(A79) $$\begin{array}{c} |H\left(Q_{UX}^{\left(\gamma \right)}\right)-H\left(P_{UX}^{\left(\gamma \right)}\right)|\leq \sqrt{\frac{2a\log2}{\gamma }}\log\frac{\left|U||X\right|}{\sqrt{\frac{2a\log2}{\gamma }}}=\Theta \left(\frac{\log n}{n^{1/4}}\right). \end{array}$$

Similar to (A79), we have

(A80) $$\begin{array}{cc} |I_{Q^{\left(\gamma \right)}}\left(U;X\right)-I_{P^{\left(\gamma \right)}}\left(U;X\right)| & \leq \Theta \left(\frac{\log n}{n^{1/4}}\right), \end{array}$$

(A81) $$\begin{array}{cc} |I_{Q^{\left(\gamma \right)}}\left(U;Y\right)-I_{P^{\left(\gamma \right)}}\left(U;Y\right)| & \leq \Theta \left(\frac{\log n}{n^{1/4}}\right). \end{array}$$

Combining (A61), (A75), (A80) and (A81), when $\gamma =\sqrt{n}$, using the definition of $R_{b,c,d}\left(\cdot \right)$ in (28), we have

$$\begin{array}{c} R_{b,c,d}\left(Q_{XYZUV}^{\left(\gamma \right)}\right) \end{array}$$

(A82) $$\begin{array}{c} \geq -\left(c+1\right)I_{Q^{\left(\gamma \right)}}\left(U;Y\right)+bI_{Q^{\left(\gamma \right)}}\left(U;X\right)-cI_{Q^{\left(\gamma \right)}}\left(V;Z\right)+dI_{Q_{\left(\gamma \right)}}\left(V;Y\right) \end{array}$$

(A83) $$\begin{array}{c} \geq -\left(c+1\right)I_{P^{\left(\gamma \right)}}\left(U;Y\right)+bI_{P^{\left(\gamma \right)}}\left(U;X\right)-cI_{P^{\left(\gamma \right)}}\left(\tilde{V};Z\right)+dI_{P^{\left(\gamma \right)}}\left(\tilde{V};Y\right)+\Theta \left(\frac{\log n}{n^{1/4}}\right) \end{array}$$

(A84) $$\begin{array}{c} =R_{b,c,d}\left(P_{XYZU\tilde{V}}^{\left(\gamma \right)}\right)+\Theta \left(\frac{\log n}{n^{1/4}}\right). \end{array}$$

The proof of Lemma A1 is now complete.
